# *Avena sativa*-derived avenanthramides suppress 12-lipoxygenase activity and downstream arachidonic acid metabolites in letrozole-induced PCOS rats

**DOI:** 10.1038/s41598-026-56474-w

**Published:** 2026-06-22

**Authors:** Yara Walid, Raghda A. Elsabbagh, Heba Handoussa, Sahar M. Abdel-Maksoud

**Affiliations:** 1 Biochemistry Department, Faculty of Pharmacy and Biotechnology, German University in Cairo (GUC), Cairo, 11835 Egypt; 2 Biochemistry Division, Medical Sciences Department, Faculty of Dentistry, German University in Cairo (GUC), Cairo, 11835 Egypt; 3 Pharmaceutical Biology, Faculty of Pharmacy and Biotechnology, German University in Cairo (GUC), Cairo, 11835 Egypt

**Keywords:** 12-lipoxygenase (12-LOX), 12-hydroxyeicosatetraenoic acid (12-HETE), Avenanthramide, Letrozole, Oats extract, Polycystic ovary syndrome (PCOS), Trans-resveratrol (RSV), Biochemistry, Diseases, Drug discovery, Endocrinology, Medical research, Physiology

## Abstract

Polycystic ovary syndrome (PCOS) implicates hormonal imbalance, ovulation disorders, metabolic disturbances, and chronic low-grade inflammation. In this study, we investigated the inflammation driven by 12-lipoxygenase (12-LOX)-mediated conversion of arachidonic acid to pro-inflammatory 12-hydroxyeicosatetraenoic acid (12-HETE) and the therapeutic potential of avenanthramide (AVA)-enriched oat extract and trans-resveratrol (RSV) as natural 12-LOX inhibitors. AVA-enriched extract was obtained from oats using 80% methanol, dried, and analyzed by HPLC. Fifty-six rats (3-week-old females) were divided into 8 groups: four received letrozole 1 mg/kg in 0.5% carboxymethyl cellulose (CMC) for 21 days to induce PCOS, while four received only CMC. Each pair (PCOS and non-PCOS) was treated for 2 weeks with either AVA (100 and 300 mg/kg), trans-resveratrol (20 mg/kg), or left untreated. Serum hormonal profile and 12-HETE levels were assessed via ELISA. Ovarian 12-LOX expression was evaluated by Western blotting. Histopathological analysis was performed on the liver and reproductive organs. Treating PCOS-induced rats with 100 mg/kg AVA, 300 mg/kg AVA, and 20 mg/kg RSV significantly restored their hormonal profile to normal levels and significantly reduced ovarian 12-LOX expression and subsequently their serum 12-HETE levels compared to the untreated PCOS rats (*P* < 0.001, *P* < 0.0001, and *P* < 0.0001, respectively). The 300 mg/kg AVA and 20 mg/kg RSV treatments restored normal ovarian and uterine architecture compared to the untreated PCOS group. No histopathological alterations were observed in the liver or oviduct. Avenanthramides from *Avena sativa* (oats) restore endocrine and ovarian function in letrozole-induced PCOS by directly inhibiting the redox-sensitive 12-lipoxygenase–12-HETE lipid signaling pathway. These findings suggest that avenanthramides may represent a promising therapeutic approach for restoring endocrine balance and ovarian function in PCOS.

**Supplementary Information:**

The online version contains supplementary material available at 10.1038/s41598-026-56474-w.

## Introduction

Polycystic Ovary Syndrome (PCOS) is a prevalent and complex endocrine disorder affecting women of reproductive age. It is characterized by hyperandrogenism, ovulatory dysfunction, and polycystic ovarian morphology. Beyond its reproductive implications, PCOS is closely associated with a cluster of metabolic disturbances, such as insulin resistance, dyslipidemia, and a state of chronic low-grade inflammation, factors that significantly elevate the risk of developing type 2 diabetes and cardiovascular disease^1,2^. In recent years, emerging literature has increasingly emphasized the role of modern dietary factors in driving the pathogenesis of PCOS. Specifically, high consumption of ultra-processed foods and artificial sweeteners, as well as high-fructose corn syrup, has been closely linked to the aggravation of both metabolic and reproductive dysfunctions in women with PCOS^3,4^. Recent studies demonstrate that excessive intake of these dietary sweeteners alters gut microbiota composition and promotes intestinal permeability. This dysbiosis triggers the systemic release of lipopolysaccharides (LPS), thereby fueling chronic low-grade inflammation through the activation of immune cells and the excessive production of reactive oxygen species (ROS)^5^. Furthermore, this diet-induced inflammatory state and subsequent hyperinsulinemia directly disrupt ovarian steroidogenesis. Elevated insulin acts synergistically with luteinizing hormone (LH) to overstimulate ovarian theca cells, upregulating steroidogenic enzymes (such as CYP17A1) and driving excessive androgen synthesis^6,7^. Consequently, dietary-driven inflammation and hyperandrogenemia create a vicious cycle that further impairs follicular maturation and ovulation. While the pathophysiology of PCOS is multifactorial, chronic inflammation is increasingly recognized as a key contributor to its endocrine and metabolic features^1,2^.

The arachidonic acid (AA) metabolic cascade is a central pathway in the generation of potent inflammatory mediators. Once released from membrane phospholipids, AA is metabolized by two major enzymatic systems: cyclooxygenases (COXs), which generate prostaglandins, and lipoxygenases (LOXs), which generate leukotrienes and hydroxyeicosatetraenoic acids (HETEs). While COX-derived prostaglandins have been extensively studied in ovarian physiology, the role of LOX pathways, particularly the 12-lipoxygenase (12-LOX) pathway, remains less clearly defined in PCOS. The 12-LOX enzyme catalyzes the conversion of AA to 12-hydroperoxyeicosatetraenoic acid (12-HPETE), which is rapidly reduced to its more stable metabolite, 12-HETE. This lipid mediator is involved in multiple pro-inflammatory and cell-signaling processes, including platelet aggregation, cell migration, and potentially steroidogenesis, suggesting it may be a critical, yet underexplored, player in the inflammatory milieu of PCOS. This leads to disturbance in the maturation of primary oocytes to secondary oocytes, which is essential for fertilization of the oocyte^8–12^. *Avena sativa* (common oat) is a rich source of unique polyphenol antioxidants known as avenanthramides (AVAs). These compounds exhibit potent anti-inflammatory and antioxidant properties, primarily through reactive oxygen species (ROS) scavenging and modulation of inflammatory signaling pathways. Emerging evidence indicates that some polyphenols can directly inhibit LOX enzymes, suggesting that AVAs may exert their anti-inflammatory effects through similar mechanisms^13–18^.

Extensive previous research emphasizes the broad therapeutic benefits of *Avena sativa* in addressing inflammatory and metabolic disorders. Studies using isolated avenanthramides, most notably the major isoforms AVA-A, AVA-B, and AVA-C, have demonstrated efficacy in suppressing pro-inflammatory cytokines such as tumor necrosis factor-alpha (TNF-α) and interleukin 6 (IL-6), downregulate nuclear factor kappa B (NF-κB) signaling, and reduce cellular oxidative stress^19,20^. Furthermore, isolated AVAs have shown significant therapeutic potential in managing metabolic syndrome, atherosclerosis, and insulin resistance^17,21^. Because these conditions share overlapping pathophysiological features with the chronic low-grade inflammation and metabolic disturbances seen in PCOS, AVAs represent a highly relevant therapeutic candidate. The justification for exploring AVAs in the study of PCOS stems from the urgent need for safer, long-term therapeutic alternatives. Conventional pharmacological interventions for PCOS, such as oral contraceptives, anti-androgens, and metformin, are frequently accompanied by adverse side effects, contraindications, or poor patient compliance^22^. AVAs, by contrast, offer a naturally derived, multi-target approach with a high safety profile. However, a notable limitation in current botanical research is the gap between using purely isolated single compounds, which provide precise pharmacokinetic data, and crude dietary extracts, which reflect realistic nutritional interventions but possess complex matrices. By utilizing an AVA-enriched extract, this study aims to bridge this gap, leveraging the natural synergistic potential and dietary relevance of oat polyphenols while directly investigating their targeted inhibition of the underexplored AA metabolism by 12-LOX inflammatory cascade in ovarian tissue.

Based on these insights, we hypothesized that an AVA-enriched extract from *Avena sativa* could ameliorate key hormonal and inflammatory abnormalities of PCOS in a letrozole-induced rat model of PCOS by directly targeting and inhibiting the 12-LOX pathway. To test this hypothesis, the present study aimed to extract an avenanthramides enriched extract from oats and analyze the AVA-enriched extract using HPLC–MS/MS. We investigated the effect of AVA-enriched extract on the serum levels of the 12-LOX metabolite,12-HETE, in PCOS-induced rats. We assessed its impact on the hormonal profile, including testosterone, estrogen, luteinizing hormone (LH), and follicle-stimulating hormone (FSH). In addition, we evaluated ovarian 12-LOX protein expression and compared the efficacy of the AVA-enriched extract to that of trans-resveratrol, a known 12-LOX inhibitor, to benchmark its therapeutic potential.

## Materials and methods

### Chemicals and reagents

Oats were purchased from a local herbalist (Cairo, Egypt). Letrozole tablets (2.5 mg, Femara®, Novartis) and trans-resveratrol capsules (100 mg capsules, Solgar, Leonia, NJ, USA) were used as received. Carboxymethyl cellulose (CMC), methanol, acetone, formalin, and petroleum ether were of analytical grade and procured from local suppliers (Power Chemical or Za Chem, Cairo, Egypt). All chemicals and reagents used were of the highest available purity.

### Preparation and analysis of avenanthramide-enriched extract

Oat grains were obtained from a local herbalist. A voucher specimen (number 00386) was deposited at the Pharmaceutical Biology Department, Faculty of Pharmacy and Biotechnology, German University in Cairo. Extraction of avenanthramides was performed using a modified protocol described by Multari et al*.*^23^. Briefly, unmilled oat was first defatted with petroleum ether. The defatted material was then extracted twice with 80% aqueous methanol (1:6 w/v) for 30 min at room temperature under continuous magnetic stirring. The combined extracts were filtered under vacuum, and the resulting filtrate was concentrated using a rotary evaporator at a temperature below 40 °C. The dried extract was reconstituted in methanol and filtered through a 0.22 µm PTFE syringe filter before analysis.

Quantitative analysis of avenanthramides was carried out using UPLC-ESI–MS/MS, according to the method described by Handoussa et al*.*, using an HPLC Agilent 1200 series instrument^24^. Phytochemical profiling and confirmation of avenanthramides were carried out using a Waters ACQUITY Xevo TQD system, consisting of an ACQUITY UPLC H-Class system and a Xevo™ TQD triple-quadrupole tandem mass spectrometer with an electrospray ionization (ESI) interface (Waters Corp., Milford, MA, USA). Chromatographic separation was achieved using an Acquity BEH C18 column (100 mm × 2.1 mm, 1.7 μm particle size). The mobile phase consisted of water containing 0.1% formic acid (A) and acetonitrile containing 0.1% formic acid (B) at a flow rate of 200 μL/min. The gradient elution started at 5% B (maintained for 1 min), increased to 100% B over 10 min (kept for 2 min), and returned to 5% B over 3 min (kept for 1 min). The MS scan was performed in positive ESI mode with the following conditions: capillary voltage 3.5 kV, cone voltage 20V, RF lens voltage 2.5V, source temperature 150 °C, and desolvation gas temperature 500 °C. Nitrogen was used as the desolvation and cone gas at flow rates of 1000 and 20 L/h, respectively. Data acquisition and system operation were controlled using MassLynx 4.1 software (Waters). Specific avenanthramides were identified using Multiple Reaction Monitoring (MRM), including the specific transition of m/z 299.6 → 146.9 for Avenanthramide-C.

### Animals

56 three-week-old female Wistar albino rats (weighing 150–200 g) (Table 1) were procured from the National Research Centre (Cairo, Egypt). The sample size was calculated using G*Power software based on ANOVA statistical testing and confirmed by previous studies^25^. The power analysis employed an alpha level of 0.05, a statistical power of 0.8, and an effect size of 0.6. Rats were housed in standard polycarbonate cages with wood-chip bedding (n = 7 rats per cage) under controlled laboratory conditions (22 ± 1 °C, 55 ± 10% humidity, 12-h light/dark cycle) with ad libitum access to a standard chow diet and water. A one-week acclimatization period preceded the experiment. Pre-established exclusion criteria included signs of severe systemic toxicity, weight loss exceeding 20% of baseline, or unexpected mortality prior to the experimental endpoint. However, all 56 animals remained healthy and completed the study protocol; therefore, no animals were excluded from the final analysis. This study was designed and is reported in accordance with the ARRIVE (Animal Research: Reporting of In Vivo Experiments) guidelines. All experimental procedures (BCH-2023-02-SMA) were approved by the Ethics Committee of the German University in Cairo (GUC) and were performed in strict accordance with the US National Institutes of Health Guide for the Care and Use of Laboratory Animals (NIH publication no. 85-23, revised 1996) GUC-00249^26^.

**Table 1:** The initial weights of the rats in all groups and the effect of different treatments on final body weight, organ weights, and fasting glucose in control and letrozole-induced PCOS rats.

Group	Initial body weight (g)	Final body weight (g)	Liver weight (g)	Reproductive organ weight (g)	Fasting glucose (mg/dL)
Ia (CMC)	191.6 ± 8.16	183 ± 19.67	6.711 ± 0.1443	1.443 ± 0.1604	100.6 ± 16.83
IIa (LTZ)	187 ± 10.42	202.7 ± 9.87	7.146 ± 0.6386	1.670 ± 0.1024	104.3 ± 13.89
Ib (CMC + 100 AVA)	188.7 ± 14.90	201.3 ± 16.26	7.043 ± 0.6879	1.519 ± 0.2111	107.7 ± 11.46
IIb (LTZ + 100 AVA)	194.6 ± 18.17	243.7 ± 23.39 a*	7.349 ± 0.8168	1.293 ± 0.2526	108.6 ± 11.27
Ic (CMC + 300 AVA)	189.6 ± 8.10	209.7 ± 12.42	6.521 ± 0.3805	1.387 ± 0.2535	111.2 ± 14.69
IIc (LTZ + 300 AVA)	187.7 ± 17.68	232.9 ± 17.53 a*	7.260 ± 0.4199	1.416 ± 0.2954	117.8 ± 16.32
Id (CMC + 20 mg/kg RSV)	188.3 ± 14.21	215.4 ± 21.72	6.024 ± 0.4970	1.603 ± 0.1329	100.3 ± 17.13
IId (LTZ + 20 mg/kg RSV)	185.0 ± 13.55	218.4 ± 14.76	6.664 ± 0.5962	1.357 ± 0.1232	113.9 ± 15.08

Reproductive organ weight includes the combined weight of the ovaries and uterus. Data are presented as mean ± SD (n = 7 rats per group). Statistical analysis was performed using one-way ANOVA test. No significant differences (*P* > 0.05) were found among the groups for initial rats’ weights, liver weight, reproductive organ weight, and fasting glucose levels. a: significant difference from CMC control group (Ia), *P < 0.05.

### Experimental design and PCOS induction

Estrous cycles were synchronized by intramuscular injection of 100 µg of estradiol valerate (Progynova®, 2 mg, Bayer, Germany) followed by 50 µg progesterone after 42 h (Protogest® 100 mg/2 mL, Marcyrl Pharmaceutical, Egypt).

Following the acclimatization period, the rats were randomly allocated into eight experimental groups (n = 7 per group) using a weight-stratified randomization approach to ensure no significant baseline differences in body weight existed among the groups (as confirmed in Table 1). While the primary investigators administering the daily oral treatments could not be blinded to group allocation due to the distinct physical nature of the formulations (e.g., CMC vehicle vs. AVA extract), all downstream biochemical assays, Western blotting, and histopathological evaluations were strictly blinded, as detailed in their respective methods sections. The study included two main groups: Control groups (Group I) and the PCOS-induced group (Group II). In the induction phase, Group I received 0.5% carboxymethyl cellulose (CMC) and Group II received 1% Letrozole (LTZ) orally daily for 21 days^27^. The estrous cycle was monitored daily via vaginal smear cytology. Smears were collected using saline-moistened cotton buds, rolled onto clean glass slides, air-dried, fixed in methanol, and stained with 1% crystal violet for 1 min before examination under a light microscope at 40 × magnification. Successful PCOS induction was confirmed by a persistent diestrus phase (Supplementary Fig. S9).

Then, the treatment phase was for 14 days. Group Ia and Group IIa did not receive any treatment, serving as negative and PCOS-induced controls, respectively. Group Ib and Group IIb received 100 mg/kg of Avenanthramide (AVA) enriched extract. Group Ic and Group IIc received 300 mg/kg of AVA-enriched extract. Group Id and Group IId received 20 mg/kg trans-resveratrol (RSV)^28^. The doses chosen for AVA-enriched extracts were made from a study by Zhang et al*.* where similar extraction was made, and the same doses were used^29^. In addition, the dose of RSV was chosen according to different studies, suggesting 20 mg/kg is the lowest dose with therapeutic effect^30,31^.

At the end of the treatment period, rats were euthanized by isoflurane overdose. Trunk blood was collected, allowed to clot, and then centrifuged at 4000 rpm for 15 min at 4 °C (Centurion, Scientific Ltd., USA). Fasting blood glucose was measured immediately from fresh serum, and the remaining serum was aliquoted and stored at − 80 °C for subsequent biochemical analysis.

**Fig. 1:**
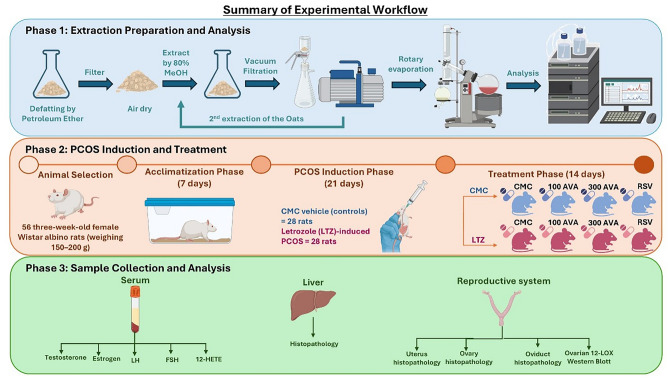
Summary of experimental workflow. Figure created in biorender.com.

The ovaries and uteri were excised, cleared of adhering fat, and weighed. One ovary from each rat was stored at − 80 °C for Western blot analysis, while the other ovary and the uterus were fixed in 10% neutral buffered formalin for histopathological examination. The experimental workflow is summarized in Fig. 1.

### Hormonal and biochemical analysis

All Enzyme-Linked Immunosorbent Assay (ELISA) kits were commercially available and used according to the manufacturers’ instructions. Serum concentrations of estrogen, testosterone, luteinizing hormone (LH), follicle-stimulating hormone (FSH), and 12-Hydroxyeicosatetraenoic Acid (12-HETE) levels were measured using the following ELISA kits (MyBioSource, Cat# MBS2607338; MyBioSource, Cat# MBS282195; LifeSpan BioSciences, Cat# LS-F27508; Elabscience, Cat# E-EL-R0391; MyBioSource, Cat# MBS268081), respectively. Regarding species reactivity, all ELISA kits were specifically validated for rat samples. According to the manufacturers’ validation data, all kits exhibit high specificity for their primary targets, with negligible cross-reactivity and no significant interference from structurally related analogs, such as other steroid hormones, glycoprotein hormones, or alternative HETE isoforms. Fasting serum glucose levels were determined using the GOD-PAP colorimetric assay (Human, Germany, Cat# 10121), and absorbance was measured spectrophotometrically at 505 nm^32^.

### Western blot analysis of ovarian 12-lipoxygenase expression

Due to assay capacity limits, a randomly selected, representative subset of ovarian tissues (n = 3 biologically independent samples per group) was utilized for Western blot analysis. Ovarian tissue samples were homogenized, and total protein was extracted using the ReadyPrep™ Protein Extraction Kit (Bio-Rad, Cat# 163-2086). Briefly, total protein was extracted, quantified using Bradford Protein Assay Kit (Bio-Basic Inc., Cat# SK3041), and equal amounts were separated by SDS-PAGE using TGX Stain-Free™ FastCast™ Acrylamide gels (Bio-Rad, Cat# 161-0181). Proteins were then transferred to a PVDF membrane using the Trans-Blot Turbo Transfer System (Bio-Rad).

Membranes were blocked with 3% Bovine Serum Albumin (BSA) in Tris-buffered saline with 0.1% Tween 20 (TBST) and then incubated with a primary antibody against 12-Lipoxygenase (1:2000 dilution; Novus Biologicals, Cat# NBP2-46512). After washing, membranes were incubated with horseradish peroxidase (HRP)-conjugated goat anti-mouse IgG secondary antibody (1:1000 dilution; Novus Biologicals, Cat# HAF007). Chemiluminescent detection was performed using Clarity™ Western ECL Substrate (Bio-Rad, Cat# 170-5060) and signals were visualized with a ChemiDoc MP Imaging System (Bio-Rad). Band intensities were quantified using ImageJ software, and 12-LOX expression was normalized to β-actin levels^33^.

### Histopathological examination

Formalin-fixed ovarian and uterine tissues were processed by standard histological procedures, including dehydration in a graded ethanol series, clearing in xylene, and embedding in paraffin wax. Sections (4 µm thick) were cut using a rotary microtome, mounted on glass slides, deparaffinized, and stained with hematoxylin and eosin (H&E) for morphological examination of tissue morphology under a light microscope^34^.

### Statistical analysis

Data analysis was performed by a researcher blinded to the treatment groups. Western blotting and histopathological assessments were performed by independent investigators who were completely blinded to the group allocations. All quantitative data were analyzed using GraphPad Prism 10.0 (GraphPad Software, CA, USA) and presented as the mean ± standard deviation (SD). Statistical comparisons between groups were made using one-way analysis of variance ANOVA followed by Tukey’s post hoc test. Statistical significance was denoted by asterisks (*P < 0.05, **P < 0.01, ***P < 0.001, ****P < 0.0001). In figures and tables, specific letters (a, b, c, d) are used in conjunction with these asterisks to denote the reference group for each statistical comparison.

## Results

### Phytochemical characterization of the avenanthramide-enriched extract

**Fig. 2:**
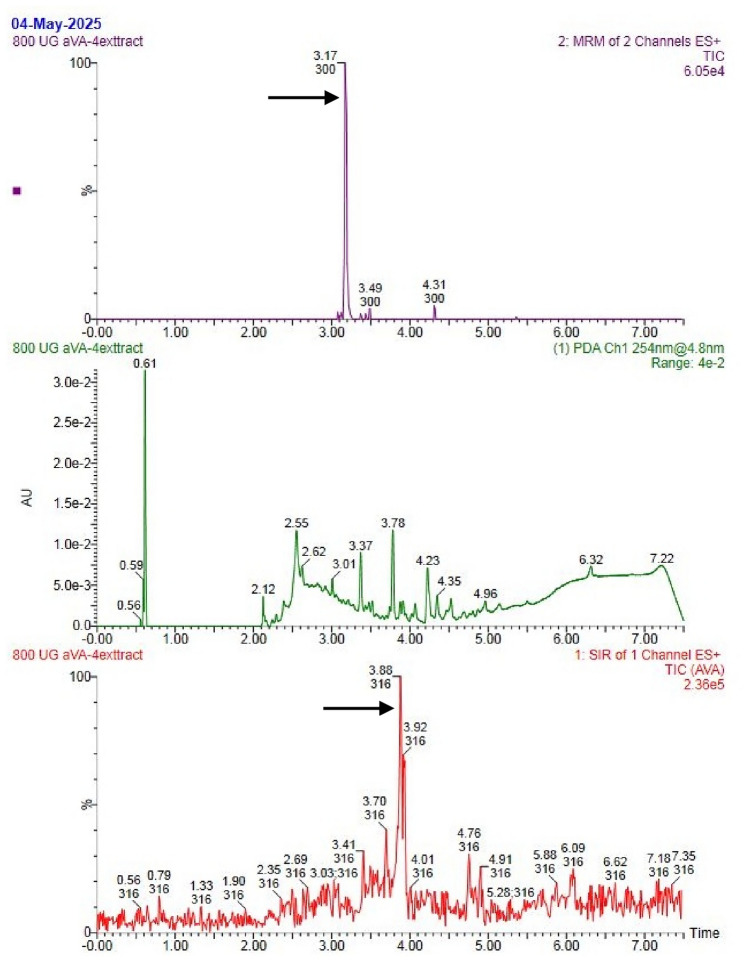
UPLC-ESI–MS/MS analysis of the Avenanthramide (AVA)-enriched extract. The representative chromatogram was acquired using Multiple Reaction Monitoring (MRM) mode, Photodiode Array (PDA) detector, and Selected Ion Recording (SIR) in positive electrospray ionization mode (ES +). The peaks confirm the presence of Avenanthramide-A (top), the PDA results of 800 µg/ml AVA-enriched extract at 254 nm wavelength (middle), and Avenanthramide-C presence in the AVA-enriched extract, identified by its specific precursor-to-product ion transition of m/z 299.6 → 146.9 (bottom).

To confirm successful enrichment of the oat extract, UPLC-ESI–MS/MS analysis was performed. Upon injection of the extract (800 µg/mL), eluted compounds were detected using a photodiode array (PDA) detector at 254 nm (Fig. 2). Further analysis using tandem mass spectrometry confirmed the presence of two specific avenanthramides (AVAs): Avenanthramide-A was identified by its mass-to-charge ratio (m/z) of 300 using Multiple Reaction Monitoring (MRM) mode, which provides high specificity and sensitivity. Avenanthramide-C was confirmed by its characteristic mass transition (m/z 299.6 → 146.9) and was identified using single ion recording (SIR) mode, which targets the detection and quantification of specific ions. These results validated the successful enrichment and identification of key AVAs in the extract (Fig. 2). Chromatographic peak areas and fragmentation patterns were utilized to profile the relative abundance of key avenanthramides in the extract, revealing a relative peak area of 8.13% for AVA-A and 47.96% for AVA-C.

### Anthropometric measures and blood glucose level

To evaluate the general health and metabolic status of the animals, key organ weights and fasting glucose levels were assessed at the end of the experimental period. No significant differences were observed in the absolute weights of the liver or the combined reproductive organs (ovaries and uterus) among any of the groups. However, the mean of final weights of the rats showed a significant increase (*P* < 0.05) in both AVA treatments with PCOS compared to the untreated non-PCOS (healthy) group. Given that these rats were in an active developmental growth phase, this weight increase likely reflects improved overall health and feed efficiency following the mitigation of inflammatory stress by AVA, potentially compounded by the well-documented anabolic effects of letrozole-induced hyperandrogenism. Fasting serum glucose levels remained within the normoglycemic range (< 126 mg/dL) across all experimental groups, with no statistically significant variation. These findings indicate that neither the letrozole-induced PCOS condition nor the subsequent treatments with AVA-enriched extract or trans-resveratrol induced notable metabolic disturbances under the present experimental design (Table 1).

### Hormonal profile

**Fig. 3:**
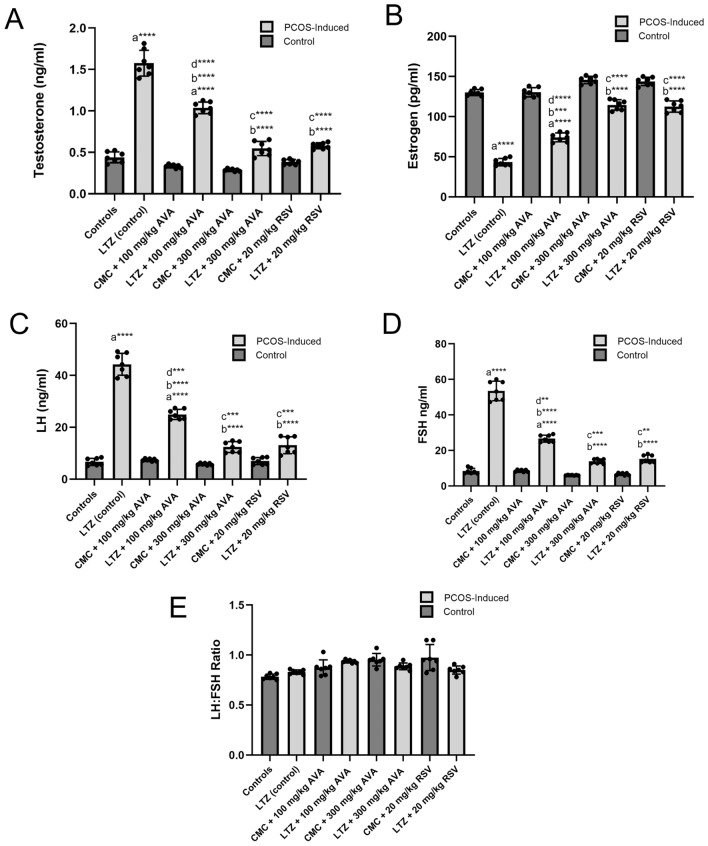
Avenanthramide (AVA)-enriched extract and 20 mg/kg trans-resveratrol (RSV) correct endocrine imbalances in letrozole-induced PCOS rats. Serum levels of (**A**) Testosterone, (**B**) Estrogen, (**C**) Luteinizing hormone (LH), (**D**) Follicle-stimulating hormone (FSH), and (**E**) LH: FSH ratio were measured in all eight experimental groups. Data are presented as mean ± SD (n = 7 biological replicates per group). Statistical analysis was performed using one-way ANOVA followed by Tukey’s multiple comparisons test. a: significant difference from CMC control group (Ia), b: significant difference from LTZ group (IIa), c: significant difference from LTZ + 100 mg/kg AVA group (IIb), d: significant difference from LTZ + 20 mg/kg RSV (IId). **** *P* < 0.0001, *** *P* < 0.001, ** *P* < 0.01, and * *P* < 0.05.

The endocrine effects of the treatment were evaluated by quantifying key reproductive hormones in serum. Letrozole administration successfully induced a hormonal profile consistent with PCOS, including significantly elevated levels of testosterone, luteinizing hormone (LH), and follicle-stimulating hormone (FSH), along with a marked suppression of estrogen compared to the healthy control group (CMC) (Fig. 3A–D).

Treatment with the AVA-enriched extract reversed these hormonal imbalances in a dose-dependent manner. The high dose (300 mg/kg AVA) significantly reduced the elevated serum testosterone levels in PCOS rats, with the same effect as that observed for trans-resveratrol (20 mg/kg RSV). The low dose AVA-enriched extract (100 mg/kg) also produced a statistically significant reduction in testosterone, though it was less pronounced compared to the healthy control group (CMC) (Fig. 3A).

Concurrently, both AVA extract doses and RSV successfully countered the letrozole-induced estrogen deficiency. High-dose AVA extract (300 mg/kg) and 20 mg/kg RSV significantly restored serum estrogen levels to values approaching those of the healthy controls. Low-dose AVA-enriched extract (100 mg/kg) also significantly increased estrogen levels, though to a lesser extent compared to the other treatments (Fig. 3B).

Letrozole-induced elevations in LH and FSH were also significantly attenuated by all treatments. Both gonadotropins were reduced by treatment with 100 mg/kg AVA, 300 mg/kg AVA, and 20 mg/kg RSV, with the 300 mg/kg AVA and 20 mg/kg RSV showing the strongest reduction effects (Fig. 3C, D). Since LH and FSH were suppressed to a similar degree, the calculated LH/FSH ratio remained unchanged across treatment groups when compared to the PCOS control (Fig. 3E).

The administration of the AVA-enriched extract or RSV to healthy, non-PCOS rats did not significantly alter their baseline hormonal profiles, indicating that the observed hormonal modulation was specific to the pathological PCOS state.

### Ovarian 12-lipoxygenase

**Fig. 4:**
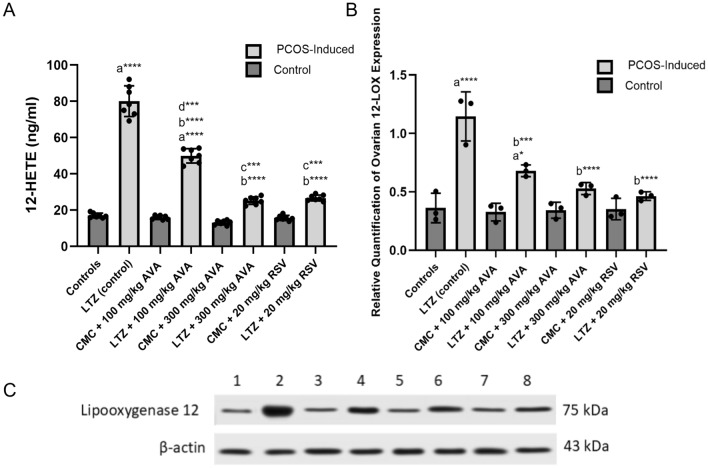
Avenanthramide (AVA)-enriched extract and 20 mg/kg trans-resveratrol (RSV) suppress 12-HETE production and ovarian 12-Lipoxygenase (12-LOX) expression in letrozole-induced PCOS rats. (**A**) Serum levels of 12-HETE were quantified by ELISA. Data are presented as mean ± SD from 7 rats per group (*n* = 7 biological replicates). (**B**) Ovarian 12-LOX protein expression was determined by Western blot. Quantification of 12-LOX protein expression was done using relative density by ImageJ, normalized to β-actin as the loading control. Data is presented as mean ± SD from 3 different rats (*n* = 3 biological replicates). (**C**) Representative western blot image. (Lanes from the same gel are shown, with cropped sections in the middle. Full-length blots are provided in the Supplementary file denoted as Fig. S1). Statistical analysis was performed using one-way ANOVA followed by Tukey’s multiple comparisons test. a: significant difference from CMC control group (Ia), b: significant difference from LTZ group (IIa), c: significant difference from LTZ + 100 mg/kg AVA group (IIb), d: significant difference from LTZ + 20 mg/kg RSV (IId). ****P < 0.0001, ***P < 0.001, **P < 0.01, and *P < 0.05. Ia: CMC control group, IIa: LTZ (PCOS control), Ib: CMC + 100 mg/kg AVA, IIb: LTZ + 100 mg/kg AVA, Ic: CMC + 300 mg/kg AVA, IIc: LTZ + 300 mg/kg AVA, Id: CMC + 20 mg/kg RSV, IId: LTZ + 20 mg/kg RSV.

To directly assess the central hypothesis of this study, we investigated the 12-lipoxygenase (12-LOX) pro-inflammatory pathway. Letrozole administration led to a significant activation of this pathway, evidenced by markedly elevated serum levels of its enzymatic product, 12-hydroxyeicosatetraenoic acid (12-HETE), compared to the healthy control group (Fig. 4A). This increase in the pro-inflammatory metabolite was supported by a significant upregulation of 12-LOX protein expression in the ovarian tissue of PCOS-control rats (Fig. 4B, C).

Treatment with the AVA-enriched extract counteracted this pathological activation in a dose-dependent manner. Both the 100 mg/kg and 300 mg/kg doses of the AVA extract, as well as trans-resveratrol (20 mg/kg RSV), significantly reduced serum 12-HETE levels and concurrently suppressed ovarian 12-LOX protein expression in PCOS rats.

Notably, the high-dose AVA extract (300 mg/kg) and 20 mg/kg RSV were the most effective in normalizing both serum 12-HETE levels and 12-LOX expression to values not significantly different from those of the healthy control group. In contrast, the 100 mg/kg AVA dose, while significantly attenuating these markers, did not achieve full normalization, as both 12-HETE and 12-LOX remained significantly elevated compared to healthy controls.

These findings strongly suggest that the therapeutic effects of the AVA-enriched extract are mediated, at least in part, by direct inhibition of the 12-LOX-mediated inflammatory signaling within the ovary.

### Histopathological analysis reveals a dose-dependent amelioration of PCOS-induced ovarian and uterine pathology by AVA-enriched extract

To evaluate the structural impact of treatment, ovarian and uterine tissues and vaginal smears were examined histologically. No pathological changes were observed in the liver or fallopian tubes of any experimental group (Supplementary Figs. S2 & S3), indicating the absence of systemic toxicity with either PCOS induction or treatment.

**Fig. 5:**
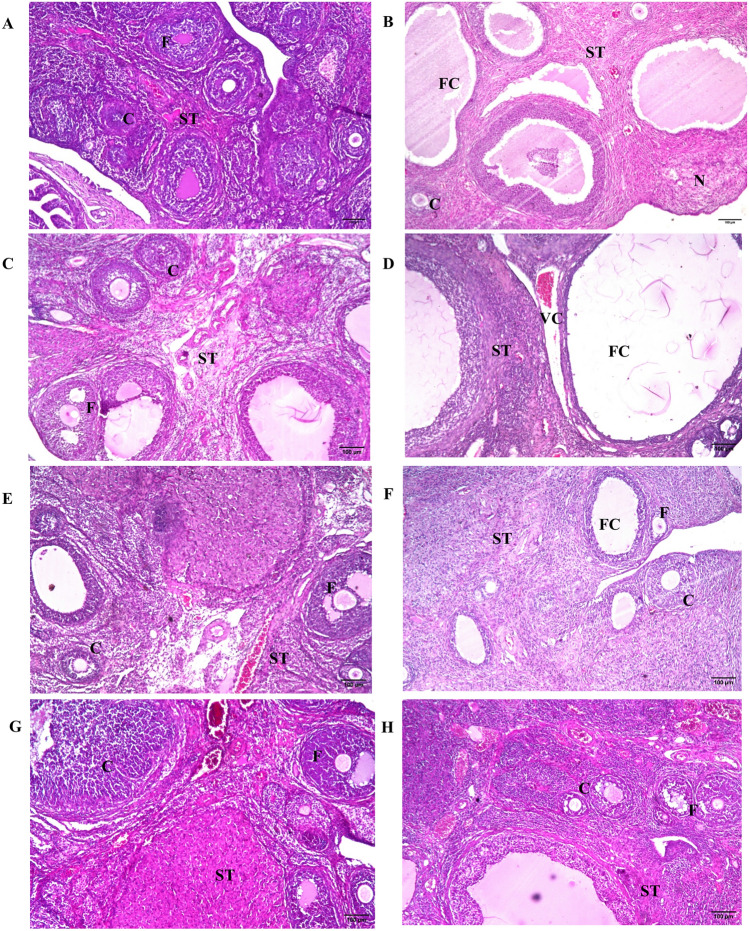
High-dose Avenanthramide (AVA)-enriched extract (300 mg/kg) and 20 mg/kg trans-resveratrol (RSV) ameliorate ovarian morphology in letrozole-induced PCOS rats (*n* = 3 biological replicates per group). Representative photomicrographs of ovarian sections stained with Hematoxylin and Eosin (H&E). (**A**) The control (CMC) group showed normal ovarian architecture with developing follicles and a corpus luteum (CL). (**B**) The Letrozole (LTZ) group exhibited multiple large follicular cysts (FC) and a lack of healthy follicles. (**C**) The CMC + AVA 100 mg/kg group showed normal histology. (**D**) The LTZ + AVA 100 mg/kg group showed persistent follicular cysts and immature follicles. (**E**) The CMC + AVA 300 mg/kg group showed normal histology. (**F**) The LTZ + AVA 300 mg/kg group showed improved follicular development with mature follicles (MF) and a CL, alongside some remaining cysts. (**G**) The CMC + 20 mg/kg RSV group showed normal histology. (H) The LTZ + 20 mg/kg RSV group showed restoration of normal ovarian structure with multiple developing follicles and CL. Scale bar = 100µm (F) graafian follicle, (FC) follicular cysts, (N) necrosis, (ST) stroma, (C) corpus luteum, (VC) vascular congestion.

**Fig. 6:**
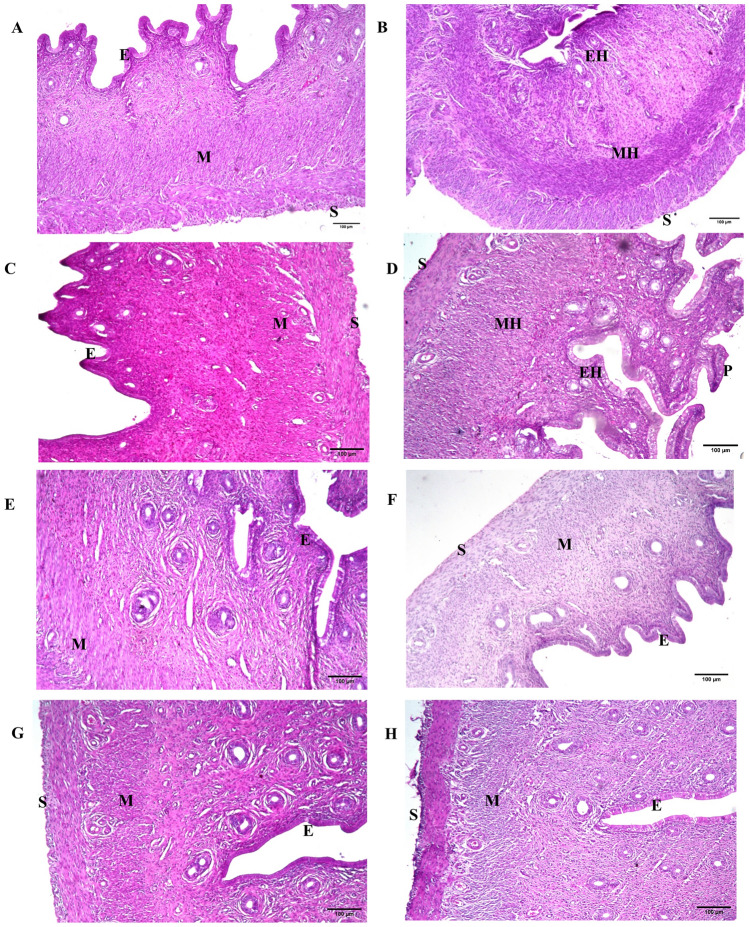
High-dose Avenanthramide (AVA)-enriched extract (300 mg/kg) and 20 mg/kg trans-resveratrol (RSV) reverse uterine pathology in letrozole-induced PCOS rats (*n* = 3 biological replicates per group). Representative photomicrographs of uterine sections stained with Hematoxylin and Eosin (H&E). (**A**) The Control (CMC) group showed a normal endometrium (En) and myometrium (My). (**B**) The letrozole (LTZ) group showed significant hypertrophy of the myometrium. (**C**) The CMC + AVA 100 mg/kg group showed a normal uterine structure. (**D**) The LTZ + AVA 100 mg/kg group showed endometrial hyperplasia, polyp formation (P), and myometrial hypertrophy. (**E**) The CMC + AVA 300 mg/kg group showed normal histology. (**F**) The LTZ + AVA 300 mg/kg group showed a complete restoration of normal uterine architecture. (**G**) The CMC + 20 mg/kg RSV group showed normal histology. (**H**) The LTZ + 20 mg/kg RSV group showed a fully restored, normal uterine structure. Scale bar = 100µm. (S) Serosa, (M) Myometrium, (E) Endometrium, (EH) Endometrial Hyperplasia, (MH) Myometrial Hypertrophy, and (P) Polyps formations.

Letrozole administration successfully induced a PCOS-like phenotype. In contrast to the control group, which exhibited ovarian histology with follicles at various maturation stages and corpora lutea (Fig. 5A), the PCOS-control (LTZ) group exhibited multiple large, atretic follicular cysts, reduced healthy developing follicles, and vascular congestion, confirming the establishment of the PCOS model (Fig. 5B and Supplementary Fig. S4). In the uterus, the control group showed normal endometrial and myometrial structure (Fig. 6A), whereas the LTZ group presented with marked hypertrophy of the myometrium (Fig. 6B).

Treatment with the low dose of AVA-enriched extract (100 mg/kg) did not reverse these pathological changes. Ovarian histology remained compromised, characterized by the persistence of follicular cysts, an abundance of immature follicles, and signs of vascular congestion (Fig. 5D and Supplementary Figs. S4 & S5). Similarly, uterine tissue from this group showed pronounced endometrial hyperplasia with polyp formation and persistent myometrial hypertrophy (Fig. 6D).

In contrast, treatment with the high dose of AVA-enriched extract (300 mg/kg) resulted in marked histological improvements. Ovaries from this group displayed multiple developing follicles and corpora lutea, with only occasional cysts and mild edema (Fig. 5F). Remarkably, uterine morphology was completely restored, showing normalized endometrial and myometrial structures, comparable to the control group (Fig. 6F and Supplementary Fig. S7).

Treatment with trans-resveratrol (20 mg/kg) produced comparable, if not slightly superior, histological restoration. The LTZ + 20mg/kg RSV group exhibited normal ovarian architecture with several healthy follicles in various maturational stages and well-formed corpora lutea, closely resembling the control group (Fig. 5H and Supplementary Fig. S6). Likewise, the uterine structure was fully restored, with no signs of hypertrophy or hyperplasia (Fig. 6H and Supplementary Fig. S8).

The administration of the AVA-enriched extract or trans-resveratrol to healthy control rats (CMC groups) did not induce any adverse histological changes in the ovaries or uteri (Figs. 5C, E, G and Fig. 6C, E, G), confirming the safety of these treatments in a non-pathological state.

**Fig. 7:**
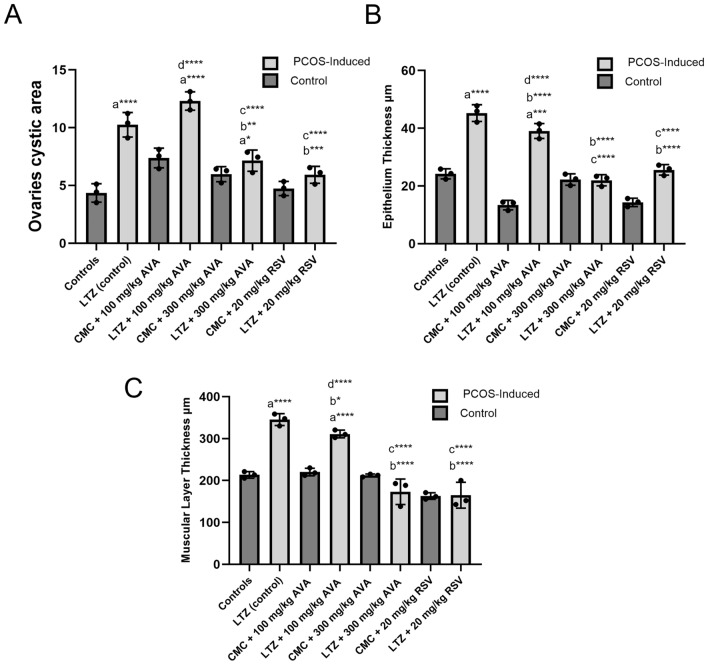
Avenanthramide (AVA)-enriched extract and 20 mg/kg trans-resveratrol (RSV) restores histopathology of the ovary and uterus in letrozole-induced PCOS rats (n = 3 biological replicates per group). (**A**) Ovarian cystic area, (**B**) Epithelium thickness, and (**C**) Muscular layer thickness were analyzed from histopathological results in all eight experimental groups using ImageJ. Data are presented as mean ± SD (n = 3 biological replicates per group). Statistical analysis was performed using one-way ANOVA followed by Tukey’s multiple comparisons test. a: significant difference from CMC control group (Ia), b: significant difference from LTZ group (IIa), c: significant difference from LTZ + 100 mg/kg AVA group (IIb), d: significant difference from LTZ + 20 mg/kg RSV (IId). ****P < 0.0001, ***P < 0.001, **P < 0.01, and *P < 0.05.

Through image interpretation and statistical analysis, the area of ovarian cysts increased significantly in the LTZ, the LTZ + 100 mg/kg AVA groups, and the LTZ + 300 mg/kg AVA group compared to the control group. However, both 300 mg/kg AVA and 20 mg/kg RSV significantly reduced cyst area compared to the LTZ group, and both treatments were more effective than low-dose AVA, as shown in Fig. 7A. Regarding the uterus, epithelial thickness increased in the LTZ and LTZ + 100 AVA groups compared to the control (CMC) group, as shown in Fig. 7B. Both treatments, high-dose AVA-enriched extract (300 mg/kg) and 20 mg/kg RSV, significantly restored the thickness to “normal” levels. In Fig. 7C, the muscular layer was significantly thicker in the LTZ group and the LTZ + 100 AVA group. Both treatments, high-dose AVA-enriched extract (300 mg/kg) and 20mg/kg RSV, restored the thickness of the muscular layer significantly. Low-dose AVA- enriched extract (100 mg/kg) was less efficient compared to the other treatments, but still significantly reduced the thickness.

## Discussion

Polycystic Ovary Syndrome (PCOS) is a prevalent endocrine disorder where chronic low-grade inflammation and oxidative stress are increasingly recognized as central contributors to its pathogenesis. Among the implicated molecular pathways, the 12-lipoxygenase (12-LOX) pathway, responsible for generating the pro-inflammatory lipid mediator 12-hydroxyeicosatetraenoic acid (12-HETE), has emerged as a critical mediator of ovarian dysfunction and metabolic disturbances in PCOS^12,35^.

A critical aspect of our findings is the clear pathological link between letrozole administration and the marked elevation of ovarian 12-LOX and serum 12-HETE. Letrozole is a potent non-steroidal aromatase inhibitor that prevents the conversion of androgens to estrogens, creating a state of profound hyperandrogenism and estrogen depletion^27,36^. Extensive literature demonstrates that hyperandrogenism directly induces mitochondrial dysfunction and severe oxidative stress, characterized by the overproduction of reactive oxygen species (ROS), within ovarian granulosa and theca cells. This androgen-induced oxidative stress acts as a critical molecular trigger. Elevated ROS levels activate redox-sensitive transcription factors, particularly nuclear factor kappa B (NF-κB), which is a known upregulator of lipoxygenase enzyme expression^37^. Simultaneously, oxidative stress activates cytosolic phospholipase A2 (cPLA2), liberating arachidonic acid from cellular membranes. Consequently, the letrozole-induced hyperandrogenic environment provides both the substrate (arachidonic acid) and the enzymatic machinery (upregulated 12-LOX via NF-κB) required to drive the massive overproduction of 12-HETE observed in our PCOS model.

In this study, we evaluated the therapeutic efficacy of an avenanthramide (AVA)-enriched oat extract, known for its anti-inflammatory properties, in a letrozole-induced PCOS rat model^13^. Trans-resveratrol (RSV), a polyphenol with established anti-inflammatory and LOX-inhibitory activity, was used as a positive control. Our primary aim was to determine whether the beneficial effects of AVAs are mediated through inhibition of the 12-LOX pathway^38^.

Histopathological analysis confirmed the successful induction of PCOS, as evidenced by multiple ovarian cysts and uterine hypertrophy in letrozole-treated rats. Treatment with high-dose AVA extract (300 mg/kg) or RSV demonstrated remarkable therapeutic efficacy and led to near-complete restoration of normal ovarian and uterine architecture. In contrast, the low-dose AVA-enriched extract (100 mg/kg) did not produce significant histological improvements. While the 100 mg/kg dose partially suppressed the 12-LOX pathway and yielded detectable improvements in serum hormone levels, these biochemical shifts were significantly weaker than those achieved with the 300 mg/kg dose. This suggests a therapeutic threshold effect, whereby a partial reduction in systemic inflammation and hyperandrogenism is insufficient to drive the complex structural remodeling and cellular repair required to reverse established ovarian cysts and uterine hypertrophy within a 14-day timeframe. These protective effects align with previous studies of AVA’s ability to ameliorate toxin-induced organ damage in the liver, as mentioned in Alwaili et al*.* and Mabrouka et al*.*^25,39^. Also, Hassanein and El-Amir demonstrated AVA’s protective effects across multiple organs, including the liver, lungs, brain, heart, kidneys, and testes, following oxidative stress induced by titanium dioxide nanoparticles^28^. This is largely attributed to avenanthramide’s antioxidant and anti-inflammatory mechanisms. Similarly, the restorative effects of 20 mg/kg RSV in our study are strongly supported by previous work by Chen et al*.* and Zhang et al*.*, who demonstrated its ability to reverse PCOS-related histopathological alteration in various rodent models^40,41^.

These structural improvements were accompanied by a profound correction of the PCOS-associated hormonal imbalance. Letrozole treatment resulted in elevated testosterone, LH, and FSH levels, alongside suppressed estrogen, mimicking key features of human PCOS. Both the high-dose AVA extract (300mg/kg) and 20 mg/kg RSV significantly reversed this hormonal dysregulation, significantly reducing androgens and gonadotropins while restoring estrogen levels. The low-dose AVA-enriched extract (100mg/kg) also elicited significant hormonal improvements, though to a lesser extent compared to the other treatments. These outcomes are supported by other studies demonstrating hormonal regulation through oat consumption in both PCOS patients and other female populations^42–44^. Moreover, the effects of 20 mg/kg RSV align perfectly with reports of its ability to normalize the hormonal milieu in PCOS models, as mentioned by Liang et al*.* and Zhang et al*.*^41,45^.

A notable finding in our study, consistent with previous literature, was the lack of a significantly altered LH: FSH ratio in the letrozole model. In human PCOS, the LH/FSH ratio is typically elevated (often > 2:1) due to rapid GnRH pulsatility that favors LH transcription, alongside the suppression of FSH by acyclic peripheral estrogens and inhibin. However, because letrozole is a potent aromatase inhibitor, it profoundly blocks the conversion of androgens to estrogens. In the rat model, this severe estrogen depletion completely removes the normal negative feedback on the hypothalamic-pituitary axis, leading to a compensatory hypersecretion of both LH and FSH. Because both gonadotropins become simultaneously elevated, the LH/FSH ratio remains unchanged. This mechanistic discrepancy is a well-documented characteristic of the oral letrozole-induced rat model, as reported by Kafali et al.^36^, and Liang et al*.*^45^. Alternative administration routes, such as continuous-release pellets, may better replicate the suppressed FSH levels seen in human PCOS, according to Maliqueo et al*.*^46^.

It is important to note that while chronic inflammation in human PCOS is tightly linked to systemic metabolic disturbances, our 21-day letrozole-induced rat model did not exhibit overt metabolic syndrome, such as fasting hyperglycemia. This aligns with previous literature indicating that short-term letrozole exposure primarily captures the endocrine and reproductive phenotypes of PCOS, whereas inducing severe metabolic features typically requires a longer induction period or a concurrent high-fat diet^47,48^. Consequently, the inflammation induced in this study was largely localized to the reproductive axis, as demonstrated by the prominent upregulation of the ovarian 12-LOX pathway and elevated serum 12-HETE. The primary sequelae of this localized inflammatory state were restricted to reproductive dysfunctions, specifically, hyperandrogenism, altered LH/FSH dynamics, and pathological alterations in ovarian and uterine architecture^48^. Therefore, the therapeutic efficacy of avenanthramides in this model highlights their capacity to resolve targeted reproductive inflammation and its local sequelae, even in the absence of severe systemic metabolic disease.

**Fig. 8:**
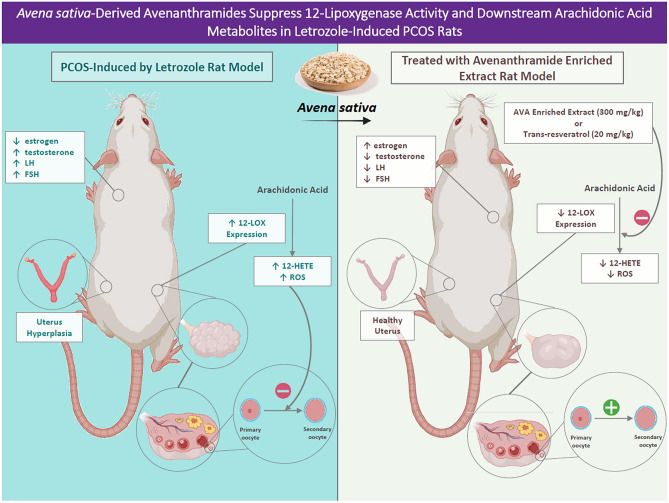
Schematic representation of the pathological cascade in the letrozole-induced PCOS rat model and the therapeutic actions of avenanthramide-enriched extract. The *left panel* illustrates the pathophysiology of a letrozole-induced rat model of Polycystic Ovary Syndrome (PCOS). This model displays key clinical features such as hyperandrogenism, ovarian cysts, uterine hyperplasia, and chronic low-grade inflammation. Pathologically, letrozole induction causes a hormonal imbalance (elevated LH, FSH, testosterone, and depleted estrogen) and increases ovarian 12-lipoxygenase (12-LOX) expression. The overexpression of 12-LOX increases the metabolism of arachidonic acid into 12-HETE, a process that generates reactive oxygen species (ROS). This, combined with hormonal and oxidative stress, disrupts follicular maturation resulting in the accumulation of immature follicles. The *right panel* demonstrates the therapeutic effects of treating the PCOS model with an Avenanthramide (AVA)-enriched extract. Treatment with 300 mg/kg AVA-enriched extract or 20 mg/kg trans-resveratrol significantly counteracts these PCOS-like changes by normalizing hormone levels and restoring healthy ovarian and uterine morphology. Mechanistically, the AVA-enriched extract inhibits the 12-LOX enzyme, thereby reducing the production of its metabolite, 12-HETE, and associated reactive oxygen species (ROS). This action corrects the impairment in follicular development, allowing for proper maturation. Figure created in biorender.com.

The central mechanistic finding of our study is the dose-dependent inhibition of the 12-LOX pathway by the AVA-enriched extract. Letrozole treatment significantly upregulated ovarian 12-LOX expression and increased serum 12-HETE levels by approximately fourfold, compared to the CMC control group (*P* < 0.0001). Treatment with the avenanthramide-enriched extract led to a marked reduction in both 12-LOX protein expression and 12-HETE concentrations, with the 300 mg/kg AVA dose restoring both parameters to levels indistinguishable from healthy controls (*P* < 0.0001 for 12-HETE levels and 12-LOX expression). This provides strong evidence that the therapeutic efficacy of AVAs is mediated, at least in part, through direct suppression of this pro-inflammatory signaling axis. This finding is strongly supported by in vitro data demonstrating that AVAs are potent LOX inhibitors^49^, with some studies suggesting superior efficacy compared to trans-resveratrol^15^. While literature directly evaluating isolated avenanthramides in in vivo PCOS models remains scarce, our findings align closely with broader studies highlighting AVA’s potent anti-inflammatory capacity in metabolic and reproductive disorders. Previous research establishes that AVAs actively attenuate inflammatory cascades by suppressing NF-κB activation and reducing downstream pro-inflammatory cytokines such as TNF-α and IL-6^19,20^. Because NF-κB acts as a key upstream transcriptional activator of lipoxygenase enzymes under oxidative stress^37^, our in vivo observation of significantly reduced ovarian 12-LOX expression provides a critical mechanistic bridge. It suggests that AVAs likely exert a dual therapeutic action in the PCOS microenvironment: directly inhibiting the 12-LOX enzyme’s catalytic activity while simultaneously suppressing the upstream transcriptional activation of the arachidonic acid inflammatory cascade. The confirmed LOX-inhibitory action of trans-resveratrol further reinforces this conclusion^15,50^ (Fig. 8).

While we did not perform a cell-free in vitro enzymatic assay in the present study to confirm direct binding to 12-LOX, previous in vitro enzyme assays have shown that avenanthramides possess direct lipoxygenase-inhibitory activity. For instance, Landberg et al.^15^ demonstrated that AVAs, particularly those containing a caffeic acid moiety, such as Avenanthramide-C (which constitutes 47.96% of our extract), significantly inhibit lipoxygenase activity in vitro. Based on this established enzyme-inhibitory capacity and our observed in vivo reduction of the 12-HETE metabolite, we infer that the therapeutic efficacy of AVAs in this model is mediated by a combination of downregulating 12-LOX expression and directly inhibiting its enzymatic activity.

Translating these preclinical findings to human application requires careful consideration of the Human Equivalent Dose (HED). Based on standard allometric scaling between species, the highly effective rat dose of 300 mg/kg translates to an HED of approximately 48.6 mg/kg. For an average 60 kg adult, this equates to a daily intake of roughly 2.9 g of the AVA-enriched extract^51^. Since native avenanthramides are present in whole oat grains and bran at relatively low concentrations, typically ranging from 10 to 300 mg/kg depending on the cultivar and processing, achieving this therapeutic dose strictly through dietary consumption of whole oats would require impractically large daily volume^20^. However, delivering 2 to 3 g of a concentrated extract is highly feasible via dietary supplements, such as capsules or functional food powders. Previous human trials utilizing AVA-enriched extracts have demonstrated excellent safety, tolerability, and bioavailability, strongly supporting the translational feasibility of AVA botanical supplements as a viable clinical adjunct for PCOS management^17^.While our findings demonstrate significant biochemical and histological improvements, a limitation of the present study is the absence of direct functional reproductive outcomes. Although we confirmed PCOS induction via the presentation of persistent diestrus, we did not quantitatively track the restoration of daily estrous cyclicity or measure exact ovulation rates (e.g., oocyte counts in the oviduct) during the treatment phase. The restoration of functional ovulation and fertility are the ultimate clinical goals in PCOS management^52^. It should be noted, however, that the histological reappearance of well-formed corpora lutea in the high-dose AVA and RSV groups provides strong indirect morphological evidence that ovulation successfully resumed. Nonetheless, to fully establish the reproductive efficacy of avenanthramides, future studies must directly evaluate these functional endpoints, including longitudinal estrous cycle tracking, oocyte quality, and successful pregnancy outcomes^47^.

Finally, we acknowledge certain limitations in our current experimental design regarding functional reproductive outcomes. The 14-day treatment duration utilized in this study was strictly optimized to evaluate immediate biochemical target engagement, specifically 12-LOX inhibition and structural tissue repair. Consequently, this short observational window did not allow for the comprehensive evaluation of long-term functional endpoints, such as the longitudinal monitoring of estrous cyclicity, exact ovulation rates (e.g., oocyte yields), mating success, or fertility rates. The restoration of functional fertility remains the ultimate clinical goal in PCOS management, and tracking these functional metrics is considered the gold standard for preclinical therapeutic evaluations^52,53^. It should be noted, however, that the histological reappearance of well-formed corpora lutea in the high-dose AVA and RSV treated groups provides strong indirect morphological evidence that the ovulatory process successfully resumed, as corpora lutea are absent in untreated letrozole-induced cystic ovaries. Nonetheless, to fully establish the long-term reproductive and fertility-restoring potential of avenanthramides, future studies utilizing extended treatment timelines are required to directly evaluate these functional endpoints. Furthermore, while UPLC-MS/MS robustly confirmed the successful enrichment of active avenanthramides in our extract, future translational studies should employ absolute quantitative standardization (e.g., precise % w/w) to facilitate exact clinical dosing.

## Conclusion

In conclusion, this study demonstrates that a high-dose avenanthramide-enriched oat extract (300 mg/kg) potently reverses the hormonal and histopathological features associated with letrozole-induced PCOS in rats. Its therapeutic efficacy, comparable to that of trans-resveratrol, appears to be mediated by suppression of the pro-inflammatory 12-LOX pathway, and therefore 12-HETE metabolite production. These findings highlight avenanthramides as promising candidates for the management of polycystic ovary syndrome and warrant further investigation into their clinical potential as natural anti-inflammatory agents targeting lipid mediator pathways.

## Supplementary Information

Supplementary Information.

## Data Availability

The authors confirm that the data supporting the findings of this study are available within the article and its supplementary materials.

## References

[1] Singh, S. et al. Polycystic ovary syndrome: Etiology, current management, and future therapeutics. *J. Clin. Med.* **12**(4), 1454 (2023). 36835989 10.3390/jcm12041454 PMC9964744

[2] Witchel, S. F., Oberfield, S. E. & Peña, A. S. Polycystic ovary syndrome: Pathophysiology, presentation, and treatment with emphasis on adolescent girls. *J. Endocr. Soc.* **3**(8), 1545–1573 (2019). 31384717 10.1210/js.2019-00078 PMC6676075

[3] Barrea, L. et al. Adherence to the mediterranean diet, dietary patterns and body composition in women with polycystic ovary syndrome (PCOS). *Nutrients* **11**(10), 2278 (2019). 31547562 10.3390/nu11102278 PMC6836220

[4] Rizk, M. G. & Thackray, V. G. Intersection of polycystic ovary syndrome and the gut microbiome. *J. Endocr. Soc.* **5**(2), bvaa177 (2021). 33381671 10.1210/jendso/bvaa177 PMC7757431

[5] Suez, J. et al. Personalized microbiome-driven effects of non-nutritive sweeteners on human glucose tolerance. *Cell* **185**(18), 3307–28.e19 (2022). 35987213 10.1016/j.cell.2022.07.016

[6] Zeng, X., Xie, Y.-J., Liu, Y.-T., Long, S.-L. & Mo, Z.-C. Polycystic ovarian syndrome: Correlation between hyperandrogenism, insulin resistance and obesity. *Clin. Chim. Acta* **502**, 214–221 (2020). 31733195 10.1016/j.cca.2019.11.003

[7] Calcaterra, V. et al. Polycystic ovary syndrome in insulin-resistant adolescents with obesity: The role of nutrition therapy and food supplements as a strategy to protect fertility. *Nutrients* **13**(6), 1848 (2021). 34071499 10.3390/nu13061848 PMC8228678

[8] Orisaka, M. et al. Chronic low-grade inflammation and ovarian dysfunction in women with polycystic ovarian syndrome, endometriosis, and aging. *Front. Endocrinol.* **14**, 1324429 (2023). 10.3389/fendo.2023.1324429 PMC10773729 38192421

[9] Zhang, Y. et al. Arachidonic acid metabolism in health and disease. *MedComm.* **4**(5), e363 (2023). 37746665 10.1002/mco2.363 PMC10511835

[10] Xu, L. et al. A genome-wide study of the lipoxygenase gene families in *Medicago truncatula* and *Medicago sativa* reveals that MtLOX24 participates in the methyl jasmonate response. *BMC Genomics* **25**(1), 195 (2024). 38373903 10.1186/s12864-024-10071-1 PMC10875803

[11] Dobrian, A. D. et al. Role of the 12-lipoxygenase pathway in diabetes pathogenesis and complications. *Pharmacol. Ther.* **195**, 100–110 (2019). 30347209 10.1016/j.pharmthera.2018.10.010 PMC6397662

[12] Ciepiela, P. et al. Arachidonic and linoleic acid derivatives impact oocyte ICSI fertilization—A prospective analysis of follicular fluid and a matched oocyte in a ‘one follicle—One retrieved oocyte—One resulting embryo’ investigational setting. *PLoS ONE* **10**(3), e0119087 (2015). 25763593 10.1371/journal.pone.0119087 PMC4357448

[13] Singh, R., De, S. & Belkheir, A. *Avena sativa* (Oat), a potential neutraceutical and therapeutic agent: An overview. *Crit. Rev. Food Sci. Nutr.* **53**(2), 126–144 (2013). 23072529 10.1080/10408398.2010.526725

[14] Gautam, R. et al. The role of lifestyle interventions in PCOS management: A systematic review. *Nutrients* **17**(2), 310 (2025). 39861440 10.3390/nu17020310 PMC11767734

[15] Landberg, R., Sunnerheim, K. & Dimberg, L. H. Avenanthramides as lipoxygenase inhibitors. *Heliyon* **6**(6), e04304 (2020). 32637696 10.1016/j.heliyon.2020.e04304 PMC7330496

[16] Jakobs, L. et al. Effects of a two-day intensive oat intake on the glucose and lipid metabolism in participants with metabolic syndrome. *Clin. Nutr. ESPEN* **46**, S775 (2021).

[17] Koenig, R. et al. Avenanthramide supplementation attenuates exercise-induced inflammation in postmenopausal women. *Nutr. J.* **13**(1), 21 (2014). 24645793 10.1186/1475-2891-13-21 PMC3999982

[18] Zhang, T. et al. Avenanthramide supplementation reduces eccentric exercise-induced inflammation in young men and women. *J. Int. Soc. Sports Nutr.* **17**(1), 41 (2020). 32711519 10.1186/s12970-020-00368-3 PMC7382060

[19] Sur, R., Nigam, A., Grote, D., Liebel, F. & Southall, M. D. Avenanthramides, polyphenols from oats, exhibit anti-inflammatory and anti-itch activity. *Arch. Dermatol. Res.* **300**(10), 569–574 (2008). 18461339 10.1007/s00403-008-0858-x

[20] Meydani, M. Potential health benefits of avenanthramides of oats. *Nutr. Rev.* **67**(12), 731–735 (2009). 19941618 10.1111/j.1753-4887.2009.00256.x

[21] Nie, L., Wise, M. L., Peterson, D. M. & Meydani, M. Avenanthramide, a polyphenol from oats, inhibits vascular smooth muscle cell proliferation and enhances nitric oxide production. *Atherosclerosis* **186**(2), 260–266 (2006). 16139284 10.1016/j.atherosclerosis.2005.07.027

[22] Legro, R. S. et al. Diagnosis and treatment of polycystic ovary syndrome: An endocrine society clinical practice guideline. *J. Clin. Endocrinol. Metab.* **98**(12), 4565–4592 (2013). 24151290 10.1210/jc.2013-2350 PMC5399492

[23] Multari, S. et al. Identification and quantification of avenanthramides and free and bound phenolic acids in eight cultivars of husked oat *Avena sativa L* from Finland. *J. Agric. Food Chem.* **66**(11), 2900–2908 (2018). 29478323 10.1021/acs.jafc.7b05726 PMC6150692

[24] Handoussa, H. et al. Anti-inflammatory and cytotoxic activities of dietary phenolics isolated from *Corchorus olitorius* and *Vitis vinifera*. *J. Funct. Foods* **5**(3), 1204–1216 (2013).

[25] Alwaili, M. A. et al. Avenanthramide-C ameliorate doxorubicin-induced hepatotoxicity via modulating Akt/GSK-3β and Wnt-4/β-catenin pathways in male rats. *Front. Mol. Biosci.* **11**, 1507786 (2024). 39687571 10.3389/fmolb.2024.1507786 PMC11646862

[26] Albus, U. Guide for the care and use of laboratory animals (8th edn). *Lab. Anim.* **46**(3), 267–268 (2012).

[27] Xu, J. et al. Letrozole rat model mimics human polycystic ovarian syndrome and changes in insulin signal pathways. *Med. Sci. Monit.* **26**, e923073 (2020). 32638705 10.12659/MSM.923073 PMC7366789

[28] Hassanein, K. M. A. & El-Amir, Y. O. Protective effects of thymoquinone and avenanthramides on titanium dioxide nanoparticles induced toxicity in Sprague-Dawley rats. *Pathol. Res. Pract.* **213**(1), 13–22 (2017). 27916297 10.1016/j.prp.2016.08.002

[29] Zhang, Y. et al. Consumption of avenanthramides extracted from oats reduces weight gain, oxidative stress, inflammation and regulates intestinal microflora in high fat diet-induced mice. *J. Funct. Foods* **65**, 103774 (2020).

[30] Juan, M. E., Vinardell, M. P. & Planas, J. M. The daily oral administration of high doses of trans-resveratrol to rats for 28 days is not harmful. *J. Nutr.* **132**(2), 257–260 (2002). 11823587 10.1093/jn/132.2.257

[31] Kumar, V. et al. Differential responses of trans-resveratrol on proliferation of neural progenitor cells and aged rat hippocampal neurogenesis. *Sci. Rep.* **6**(1), 28142 (2016). 27334554 10.1038/srep28142 PMC4917886

[32] Shaker, G. & Zubair, M. Peroxidase-Coupled Glucose Method StatPearls [Internet]: Treasure Island (FL): StatPearls Publishing; [updated 2025 Jun 2. Available from: https://www.ncbi.nlm.nih.gov/books/NBK594277/ (2025). 37603668

[33] Al-Shabrawey, M. et al. Increased expression and activity of 12-lipoxygenase in oxygen-induced ischemic retinopathy and proliferative diabetic retinopathy. *Diabetes* **60**(2), 614–624 (2011). 21228311 10.2337/db10-0008 PMC3028363

[34] Banchroft, J. D., Stevens, A. & Turner, D. R. *Theory and Practice of Histological Techniques*. Fourth Edition ed: Churchil Livingstone, New York, London, San Francisco, Tokyo (1996).

[35] Szczuko, M. et al. The role of arachidonic and linoleic acid derivatives in pathological pregnancies and the human reproduction process. *Int. J. Mol. Sci.* **21**(24), 9628 (2020). 33348841 10.3390/ijms21249628 PMC7766587

[36] Kafali, H., Iriadam, M., Ozardalı, I. & Demir, N. Letrozole-induced polycystic ovaries in the rat: a new model for cystic ovarian disease. *Arch. Med. Res.* **35**(2), 103–108 (2004). 15010188 10.1016/j.arcmed.2003.10.005

[37] Kuhn, H., Banthiya, S. & Van Leyen, K. Mammalian lipoxygenases and their biological relevance. *Biochim. Biophys. Acta BBA Mol. Cell Biol. Lipids* **1851**(4), 308–330 (2015). 10.1016/j.bbalip.2014.10.002 PMC4370320 25316652

[38] Salehi, B. et al. Resveratrol: A double-edged sword in health benefits. *Biomedicines.* **6**(3), 91 (2018). 30205595 10.3390/biomedicines6030091 PMC6164842

[39] Ltaif, M., Gargouri, M. & Soussi, A. Protective effects of *A. sativa* against oxidative stress‐induced liver damage in ovariectomized mice. *Biomed. Res. Int.* **2021**(1), 1–15 (2021). 34337029 10.1155/2021/5577498 PMC8298160

[40] Chen, M. et al. Resveratrol ameliorates polycystic ovary syndrome via transzonal projections within oocyte-granulosa cell communication. *Theranostics.* **12**(2), 782–795 (2022). 34976213 10.7150/thno.67167 PMC8692920

[41] Zhang, N. et al. Beneficial phytoestrogenic effects of resveratrol on polycystic ovary syndromein rat model. *Gynecol. Endocrinol.* **37**(4), 337–341 (2021). 32851887 10.1080/09513590.2020.1812569

[42] Friedrich, M. The effects of diet enrichment with a hull-less oat cultivar in glucose, lipid, lipoprotein, fibrinogen, and estradiol contents in the blood of post-mastectomy women. *Pol. J. Food Nutr. Sci.* **53**(4), 69–74 (2003).

[43] Mei, S., Ding, J., Wang, K., Ni, Z. & Yu, J. Mediterranean diet combined with a low-carbohydrate dietary pattern in the treatment of overweight polycystic ovary syndrome patients. *Front. Nutr.* **9**, 876620 (2022). 35445067 10.3389/fnut.2022.876620 PMC9014200

[44] Shishehgar, F., Mirmiran, P., Rahmati, M., Tohidi, M. & Ramezani Tehrani, F. Does a restricted energy low glycemic index diet have a different effect on overweight women with or without polycystic ovary syndrome?. *BMC Endocr. Disord.* **19**(1), 93 (2019). 31477085 10.1186/s12902-019-0420-1 PMC6720418

[45] Liang, A. et al. Resveratrol improves follicular development of PCOS rats by regulating the glycolytic pathway. *Mol. Nutr. Food Res.* **65**(24), 2100457 (2021). 10.1002/mnfr.202100457 34664388

[46] Maliqueo, M. et al. Continuous administration of a P450 aromatase inhibitor induces polycystic ovary syndrome with a metabolic and endocrine phenotype in female rats at adult age. *Endocrinology* **154**(1), 434–445 (2013). 23183180 10.1210/en.2012-1693

[47] Ryu, Y., Kim, S. W., Kim, Y. Y. & Ku, S.-Y. Animal models for human polycystic ovary syndrome (PCOS) focused on the use of indirect hormonal perturbations: A review of the literature. *Int. J. Mol. Sci.* **20**(11), 2720 (2019). 31163591 10.3390/ijms20112720 PMC6600358

[48] Torres, P. J. et al. Letrozole treatment of adult female mice results in a similar reproductive phenotype but distinct changes in metabolism and the gut microbiome compared to pubertal mice. *BMC Microbiol.* **19**(1), 57 (2019). 30871463 10.1186/s12866-019-1425-7 PMC6419356

[49] Ratnasari, N., Walters, M. & Tsopmo, A. Antioxidant and lipoxygenase activities of polyphenol extracts from oat brans treated with polysaccharide degrading enzymes. *Heliyon* **3**(7), e00351 (2017). 28725872 10.1016/j.heliyon.2017.e00351 PMC5506884

[50] Chatterjee, M., Das, S., Janarthan, M., Ramachandran, H. K. & Chatterjee, M. RETRACTED: Role of 5-lipoxygenase in resveratrol mediated suppression of 7,12-dimethylbenz(α)anthracene-induced mammary carcinogenesis in rats. *Eur. J. Pharmacol.* **668**(1–2), 99–106 (2011). 21749863 10.1016/j.ejphar.2011.06.039

[51] Nair, A. & Jacob, S. A simple practice guide for dose conversion between animals and human. *J. Basic Clin. Pharm.* **7**(2), 27 (2016). 27057123 10.4103/0976-0105.177703 PMC4804402

[52] Walters, K. A., Allan, C. M. & Handelsman, D. J. Rodent models for human polycystic ovary syndrome1. *Biol. Reprod.* **86**(5), 149 (2012). 22337333 10.1095/biolreprod.111.097808

[53] Legro, R. S. et al. Letrozole versus clomiphene for infertility in the polycystic ovary syndrome. *N. Engl. J. Med.* **371**(2), 119–129 (2014). 25006718 10.1056/NEJMoa1313517 PMC4175743

