# Supplementary material for: Avena sativa-derived avenanthramides suppress 12-lipoxygenase activity and downstream arachidonic acid metabolites in letrozole-induced PCOS rats
Source: Sci Rep. 2026 Jun 22;16:19345. doi: 10.1038/s41598-026-56474-w (PMC13287617; doi:10.1038/s41598-026-56474-w)
Supplement: Supplementary file 1 — Supplementary Information. [file 41598_2026_56474_MOESM1_ESM.docx]

**Full Length Western Blot Images**

For Figure 4C:


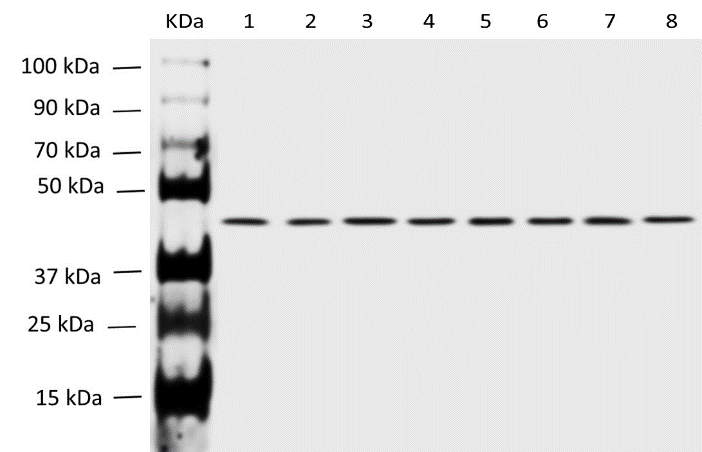

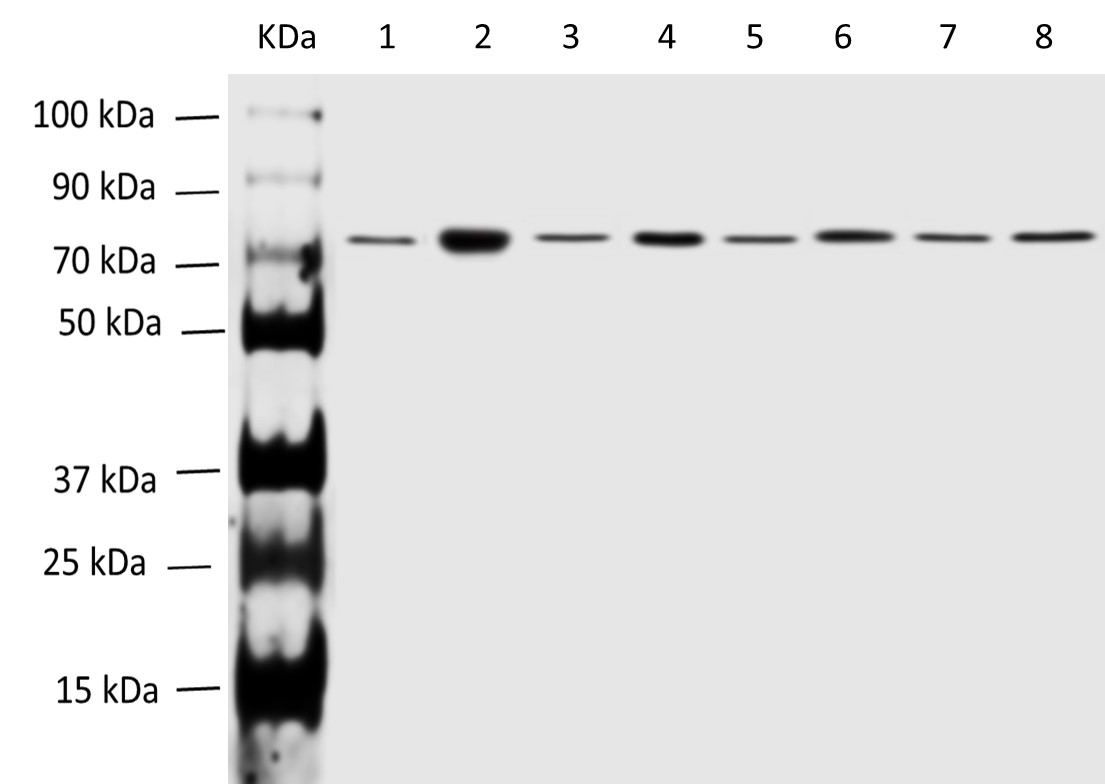


**Figure S1. Representative western blot image showing the expression of 12-Lipoxygenase in the ovaries on the right and Beta-actin on the left.**


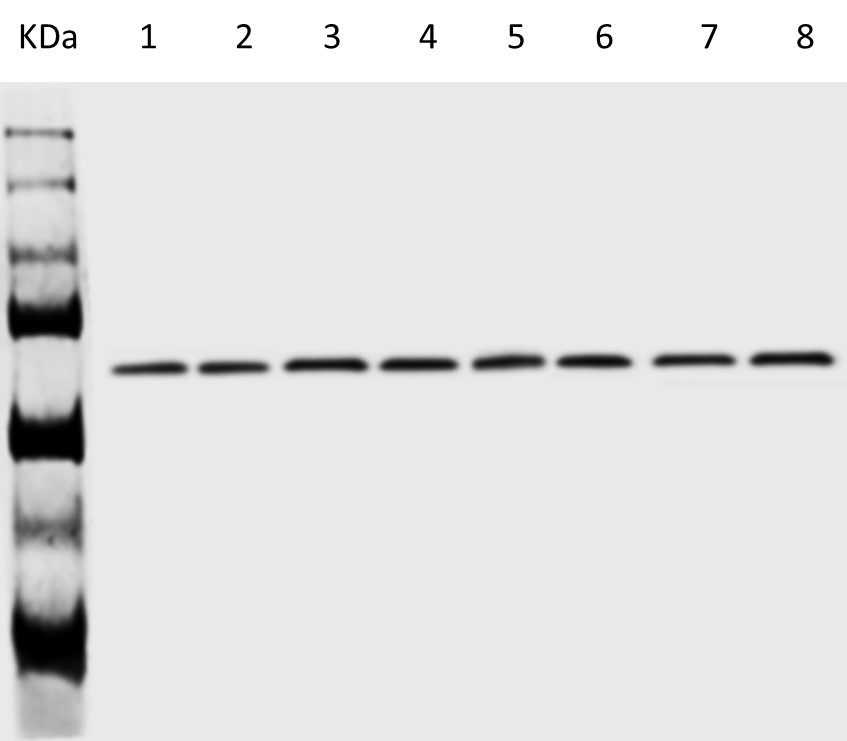

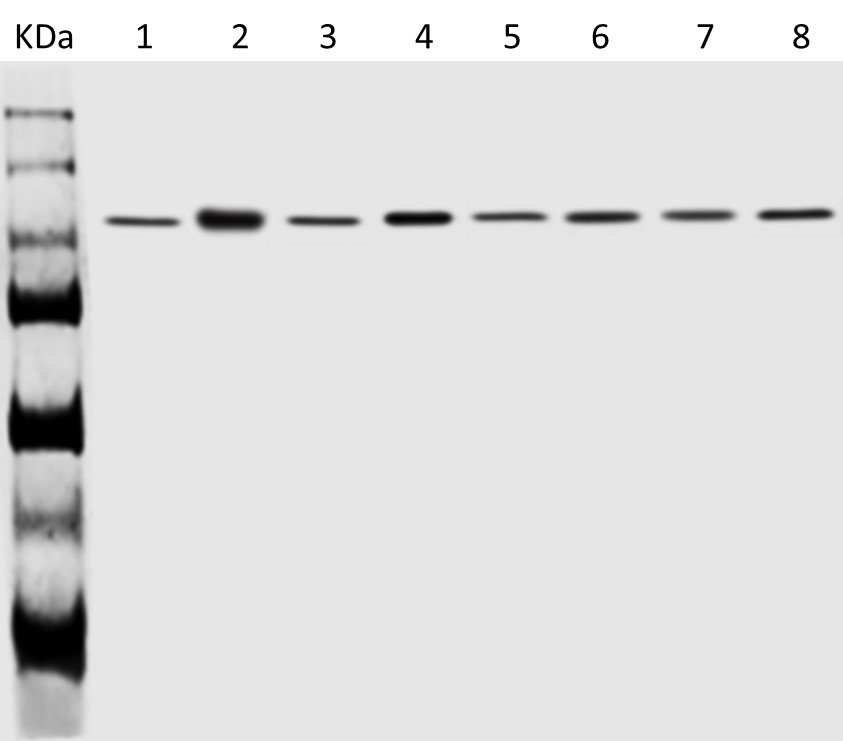

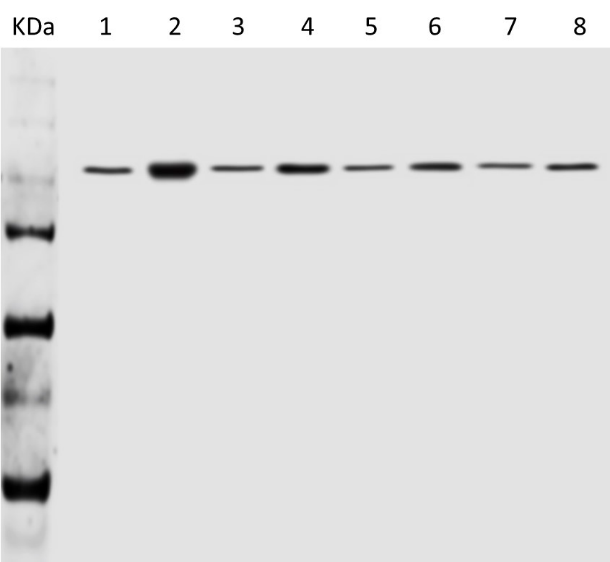

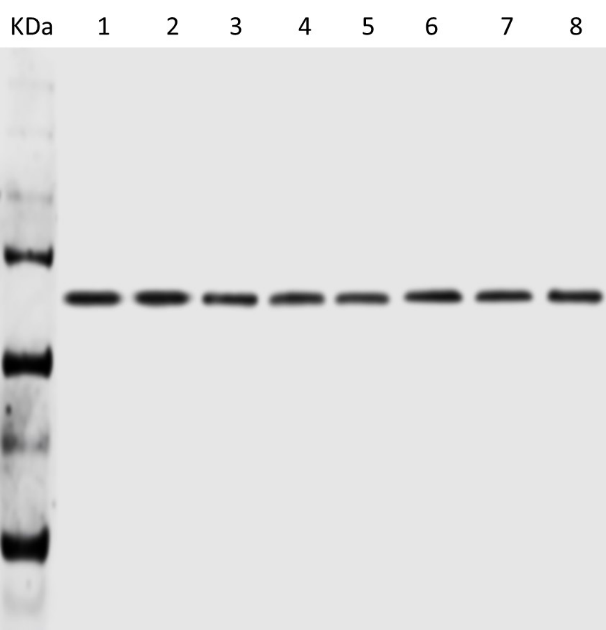


**Histopathology pictures**

Oviducts (Fallopian Tubes):


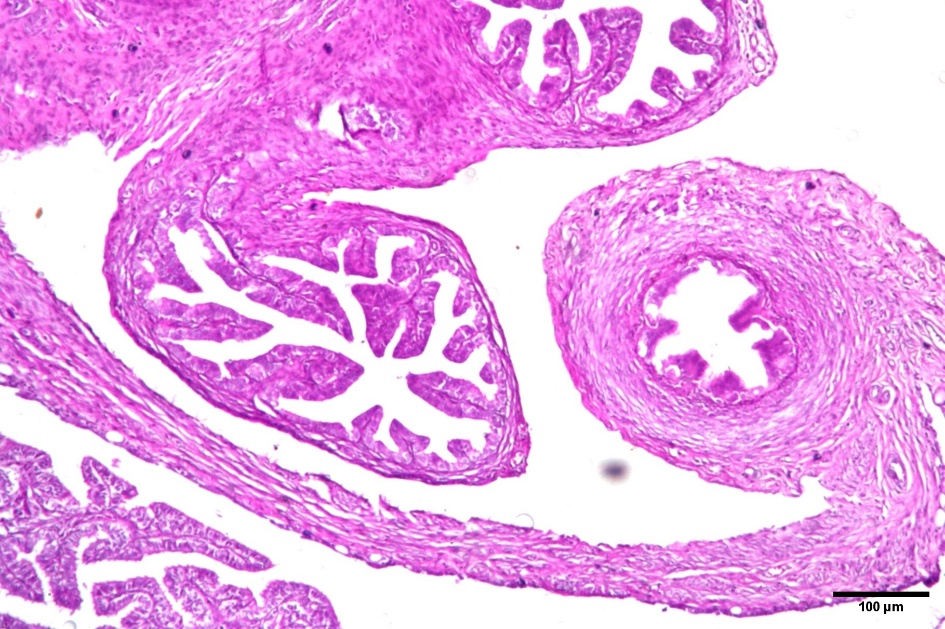

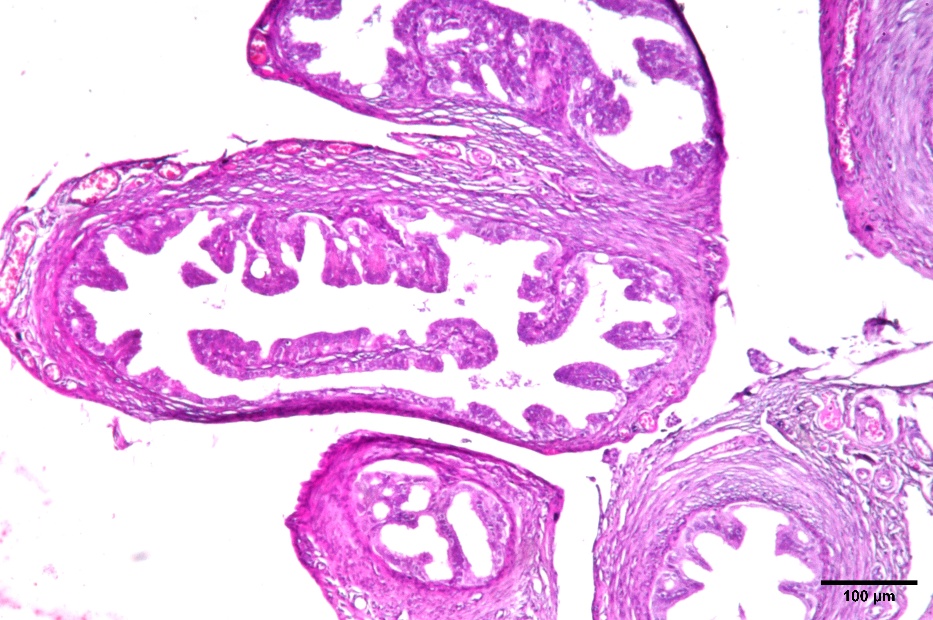

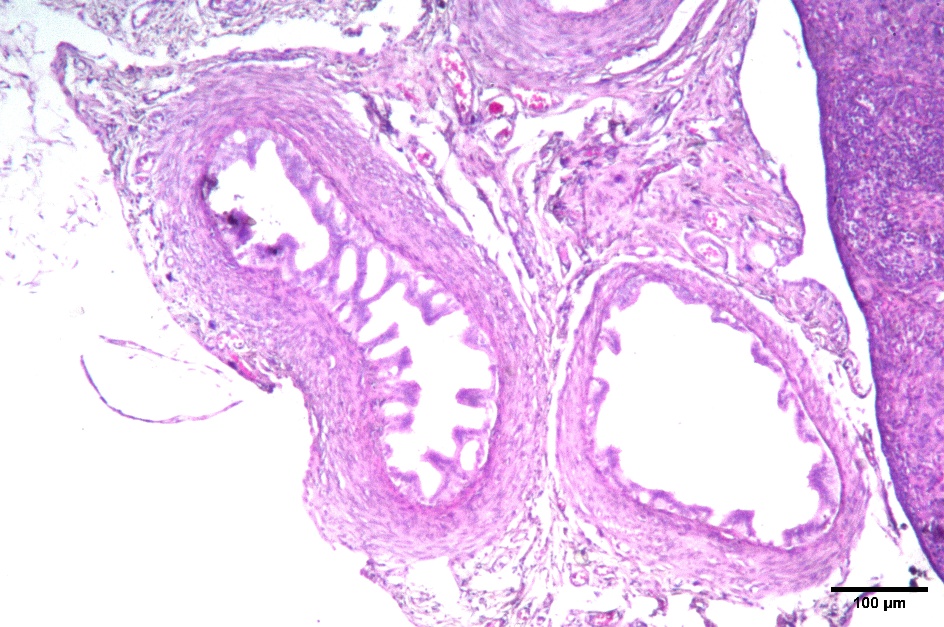

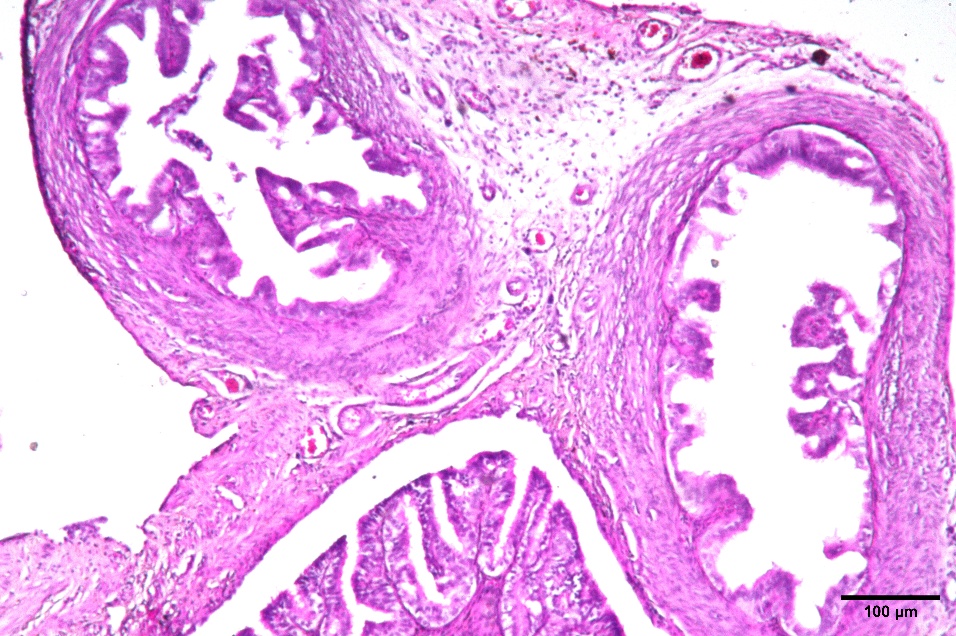

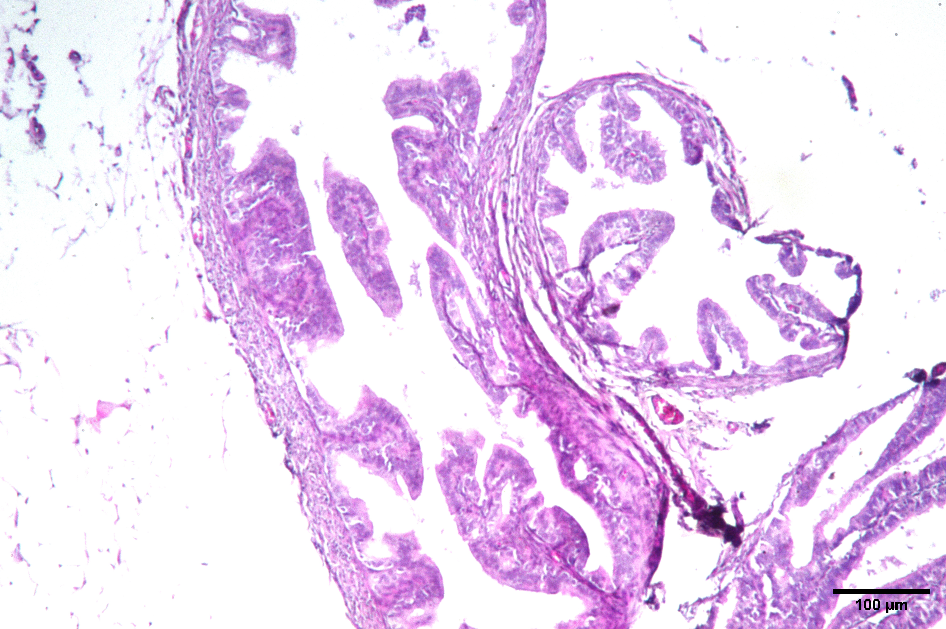

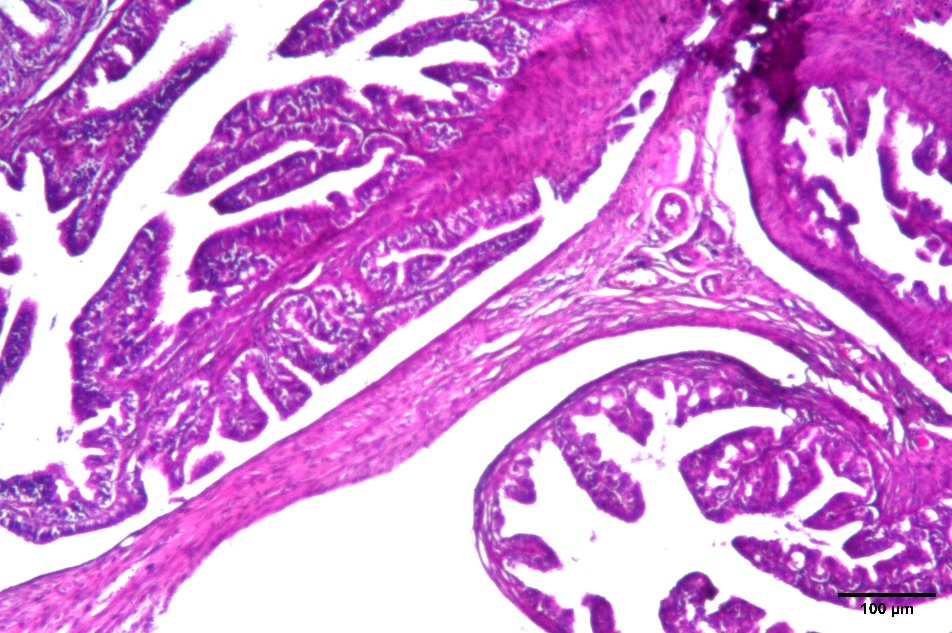

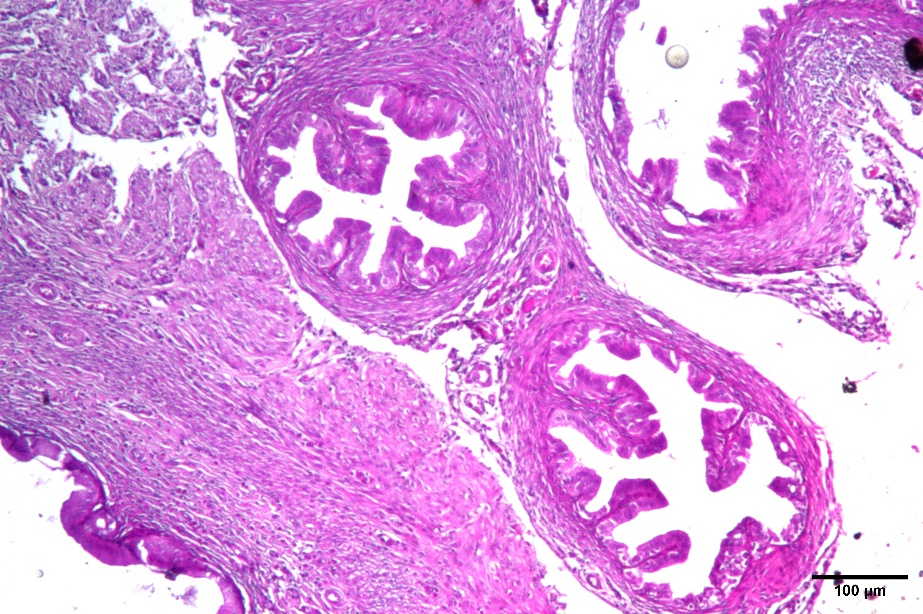


**A**

**B**

**C**

**D**

**E**

**F**

**G**

**H**


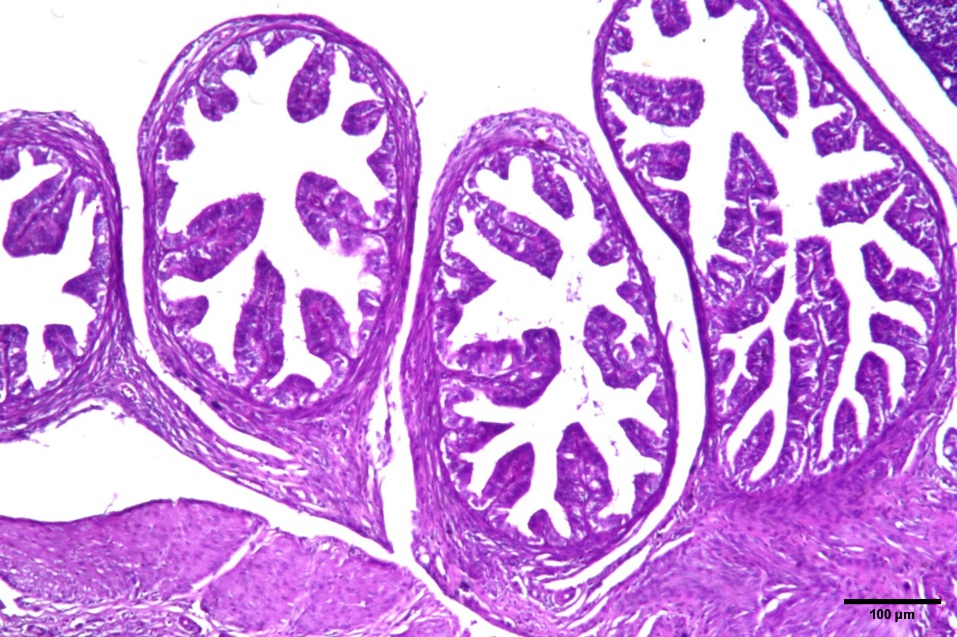


**Figure S2** **Representative photomicrographs of oviducts (fallopian tubes) sections stained with Hematoxylin and Eosin (H&E). (A)** Control (CMC) group **(B)** Letrozole (LTZ) group **(C)** CMC + AVA 100 mg/kg group **(D)** LTZ + AVA 100 mg/kg group **(E)** CMC + AVA 300 mg/kg group. **(F)** LTZ + AVA 300 mg/kg group **(G)** CMC + RSV 20 mg/kg group **(H)** LTZ + RSV 20 mg/kg group. (Magnification 20x, Scale bar = 100µm).


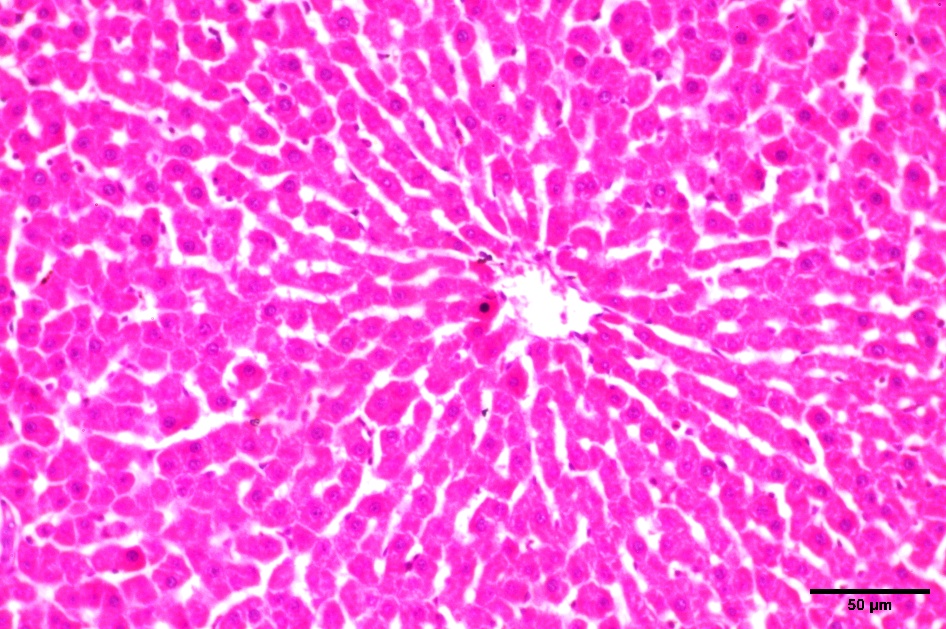

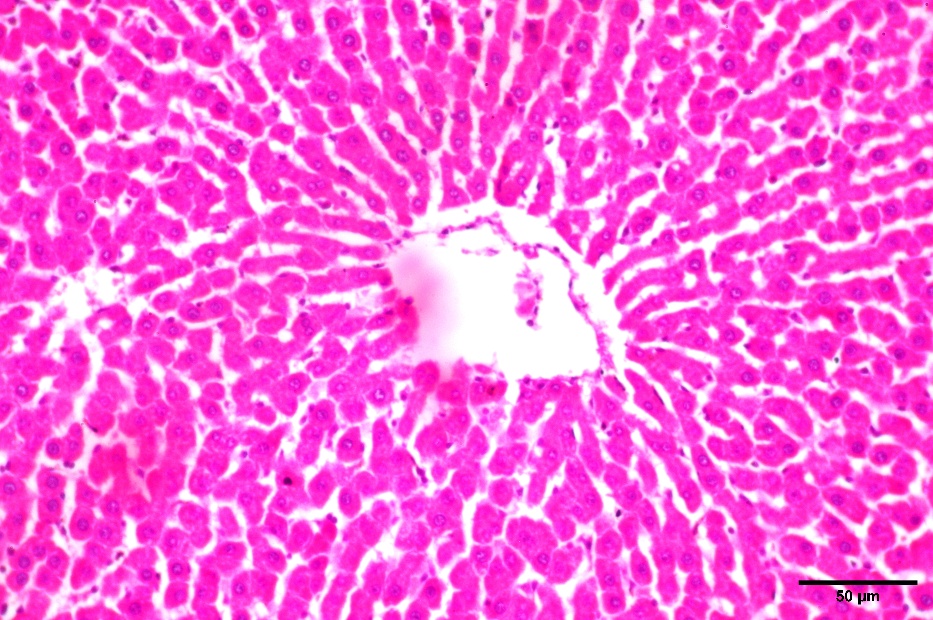

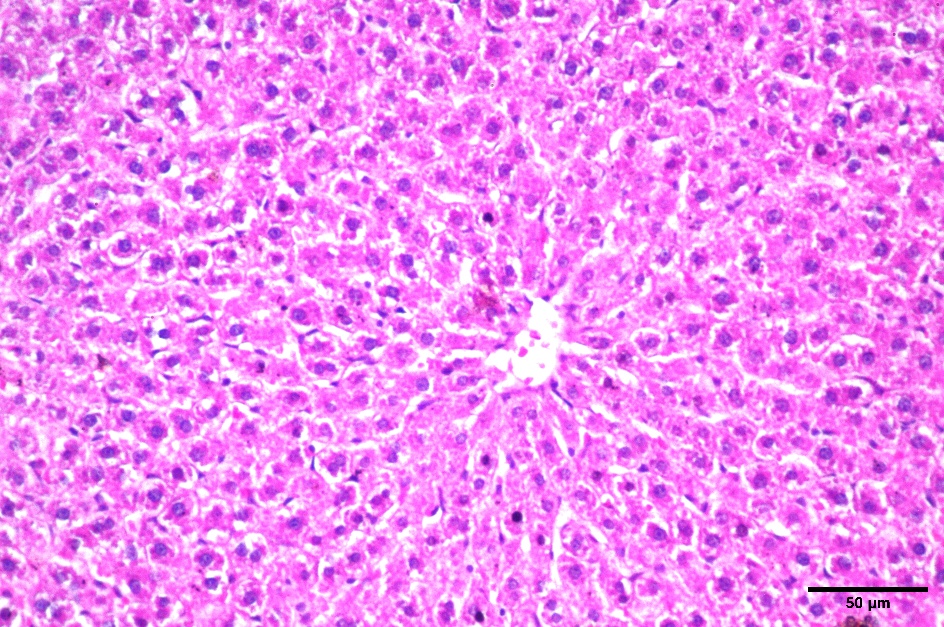

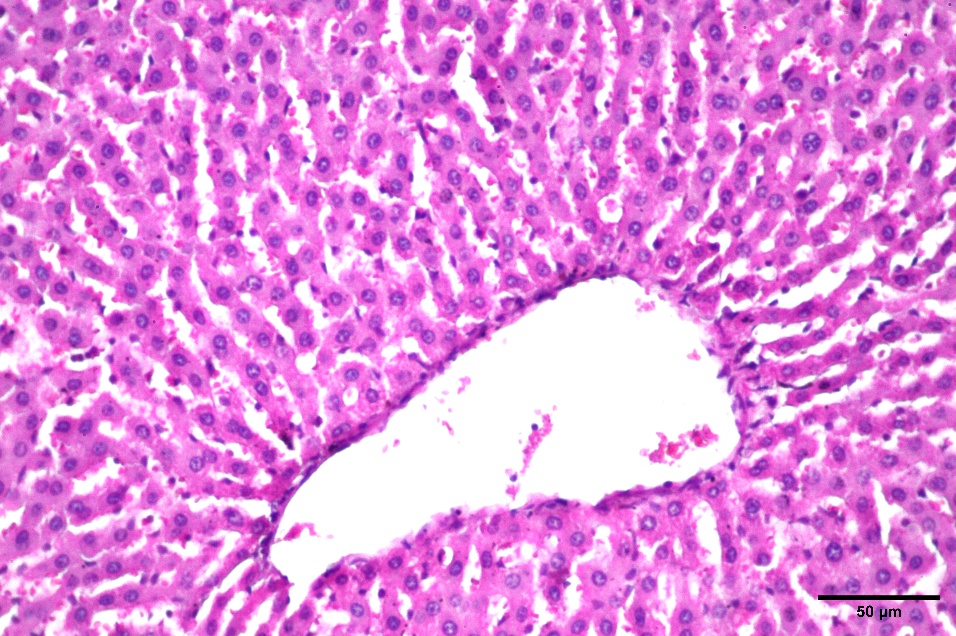

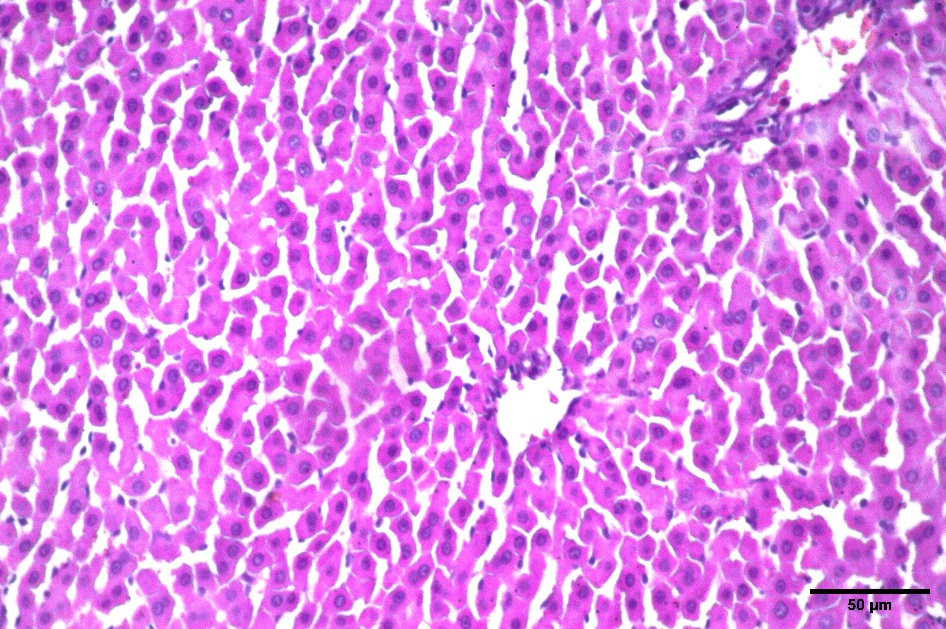

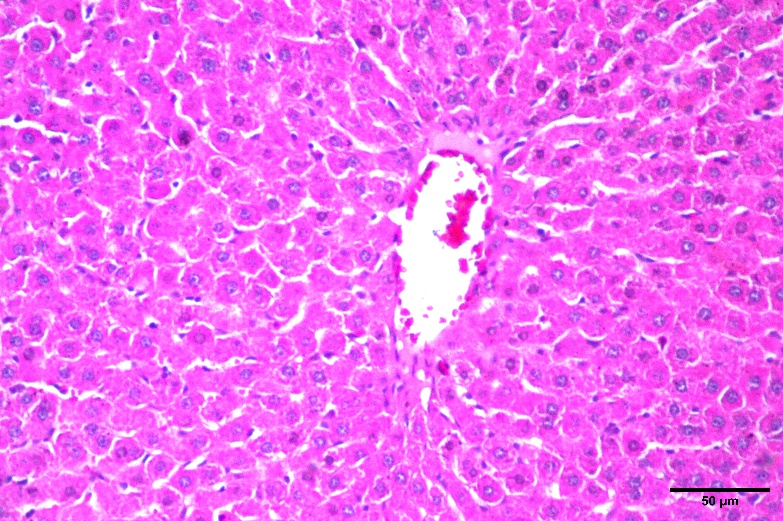

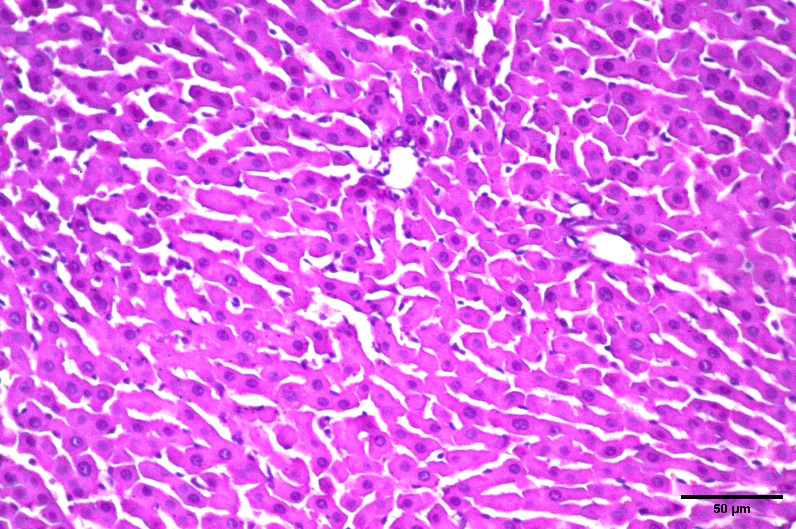


**A**

**B**

**C**

**D**

**E**

**F**

**G**

**H**


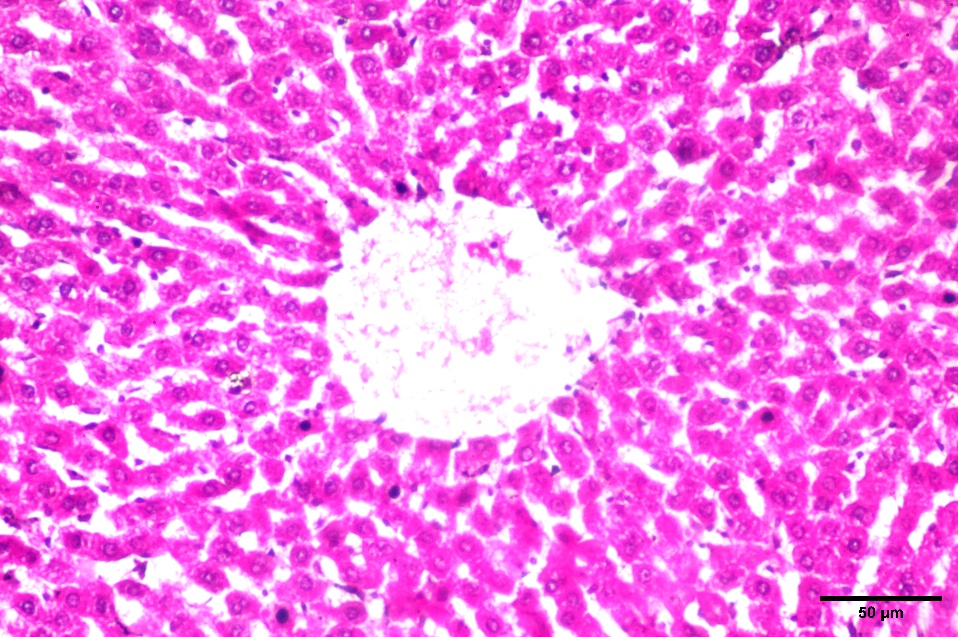


**K**


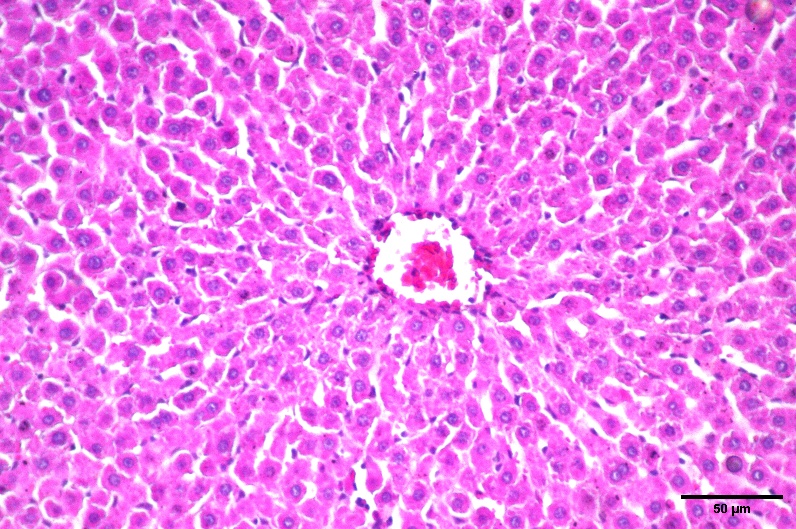


**Figure S8 Representative photomicrographs of liver sections stained with Hematoxylin and Eosin (H&E). (A)** Control (CMC) group **(B & C)** Letrozole (LTZ) group **(D)** CMC + AVA 100 mg/kg group **(E)** LTZ + AVA 100 mg/kg group **(F)** CMC + AVA 300 mg/kg group. **(G)** LTZ + AVA 300 mg/kg group **(H)** CMC + RSV 20 mg/kg group **(K)** LTZ + RSV 20 mg/kg group. (Magnification 40x, Scale bar = 50µm).


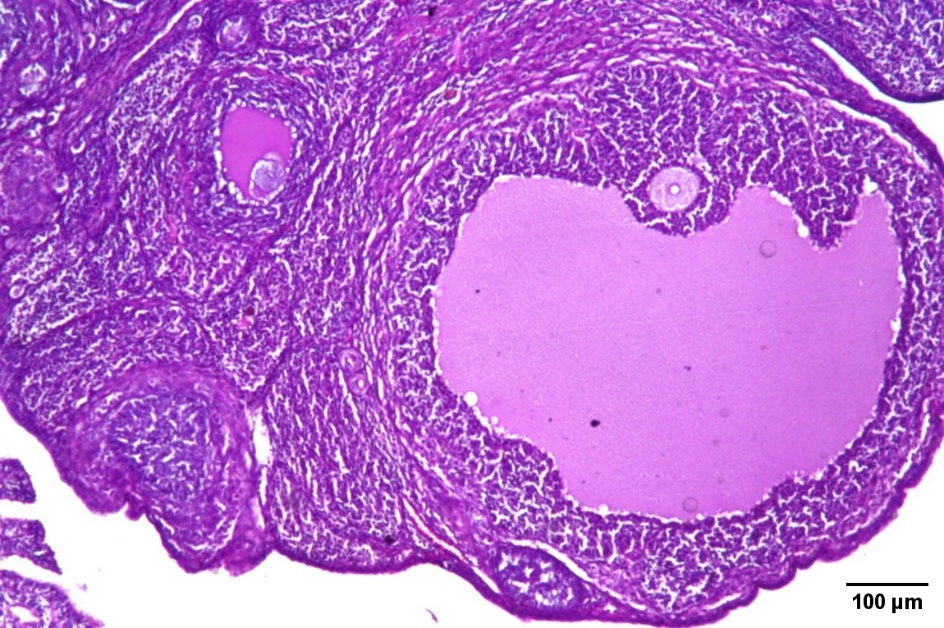

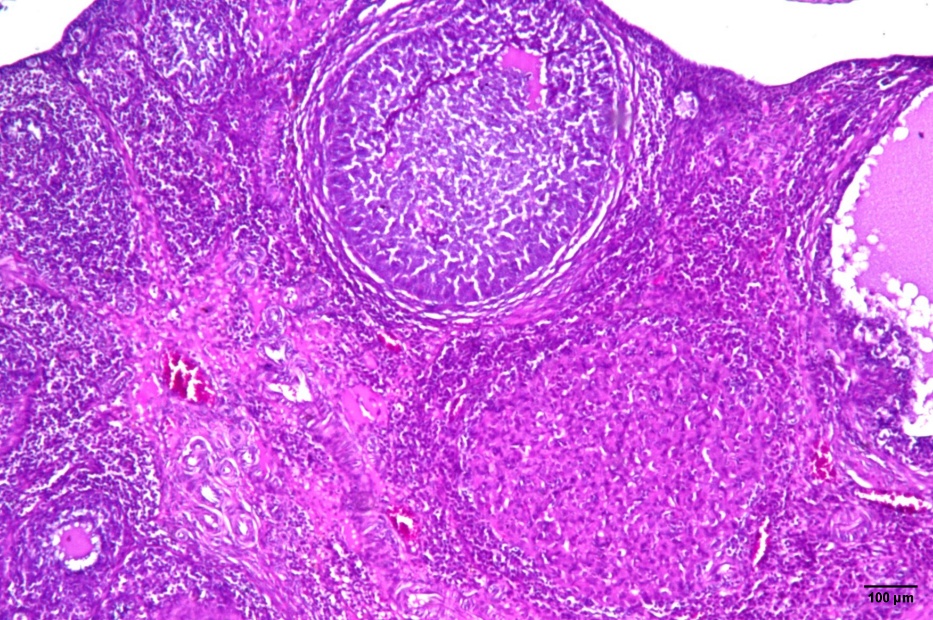

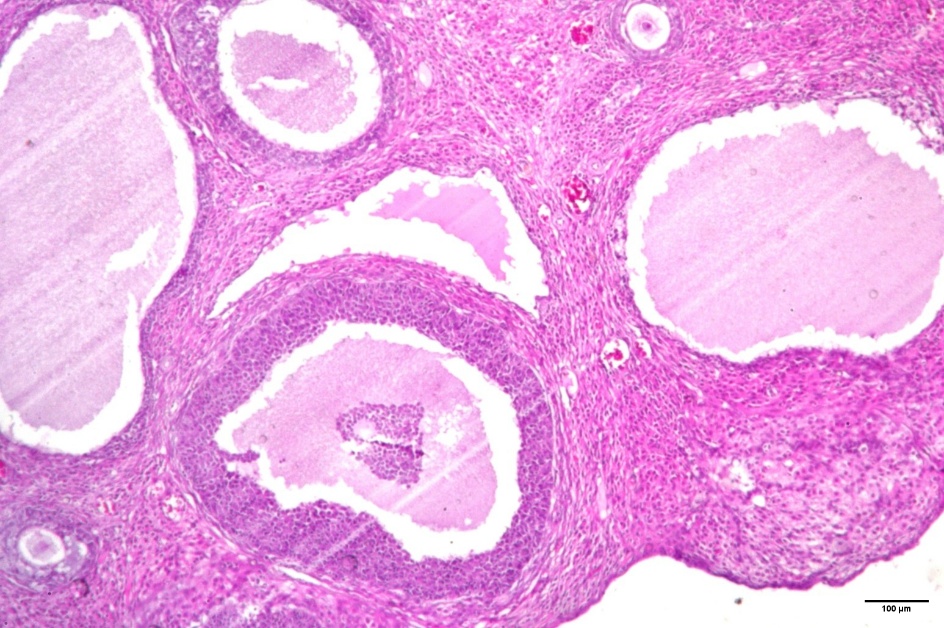

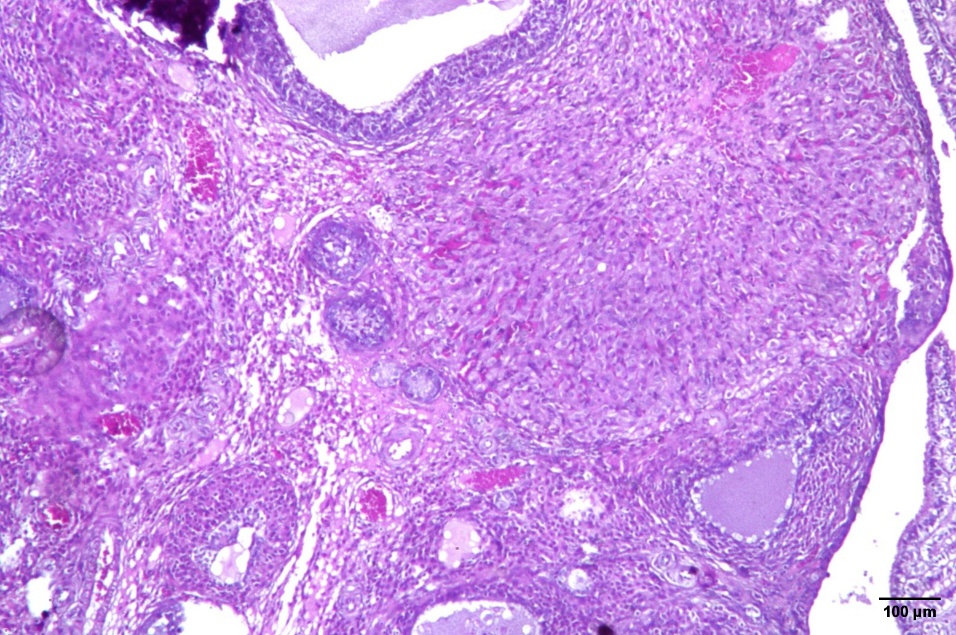

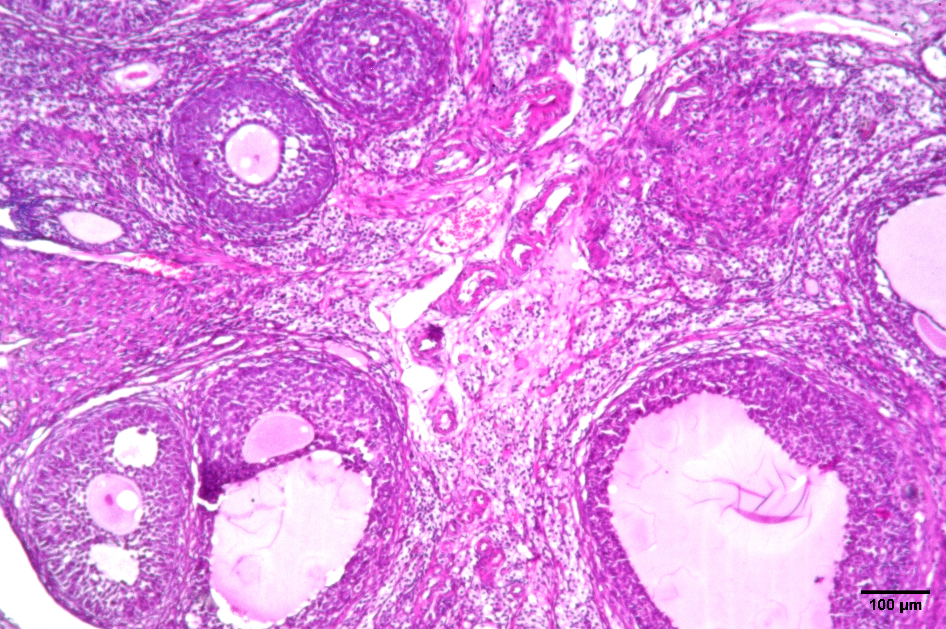

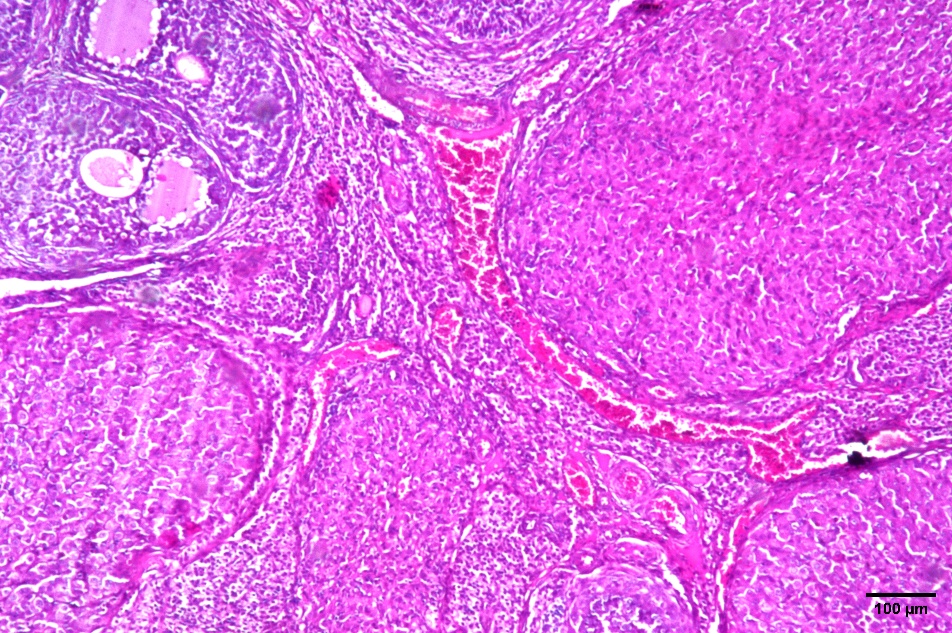

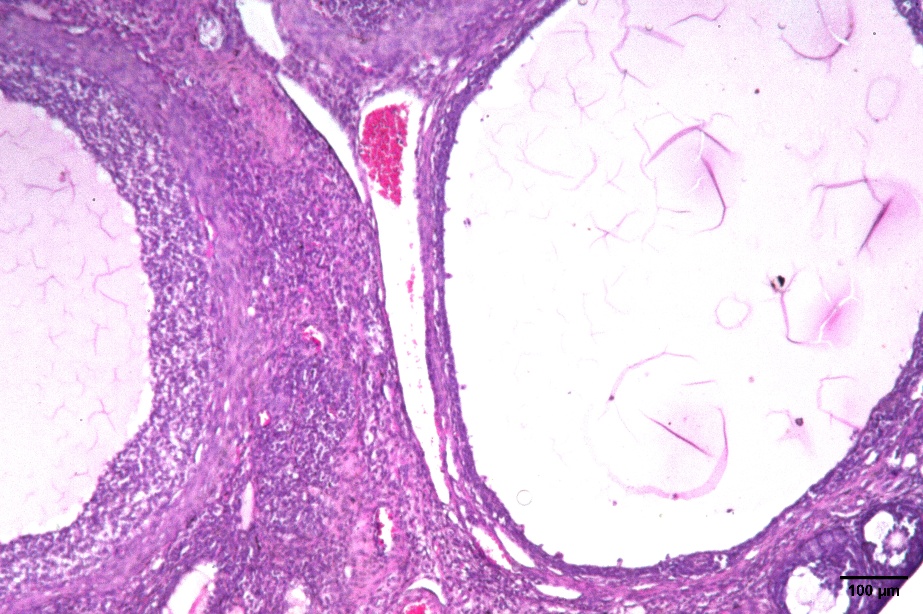


**A**

**B**

**C**

**D**

**E**

**F**

**G**

**H**


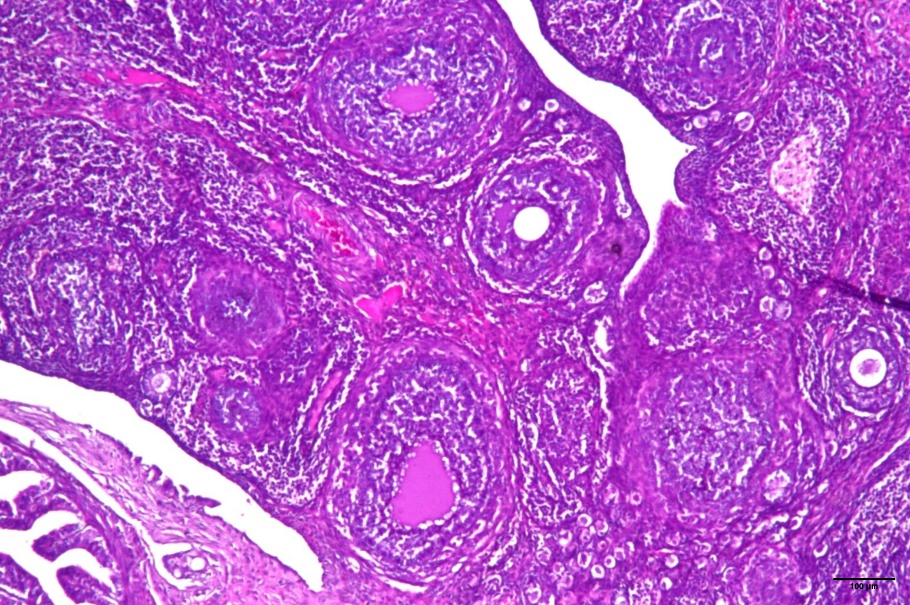


**Figure S4. Representative photomicrographs of ovarian sections stained with Hematoxylin and Eosin (H&E). (A, B, & C)** Control (CMC) group **(D & E)** Letrozole (LTZ) group **(F & G)** CMC + AVA 100 mg/kg group **(H)** LTZ + AVA 100 mg/kg group. Scale bar = 100µm.


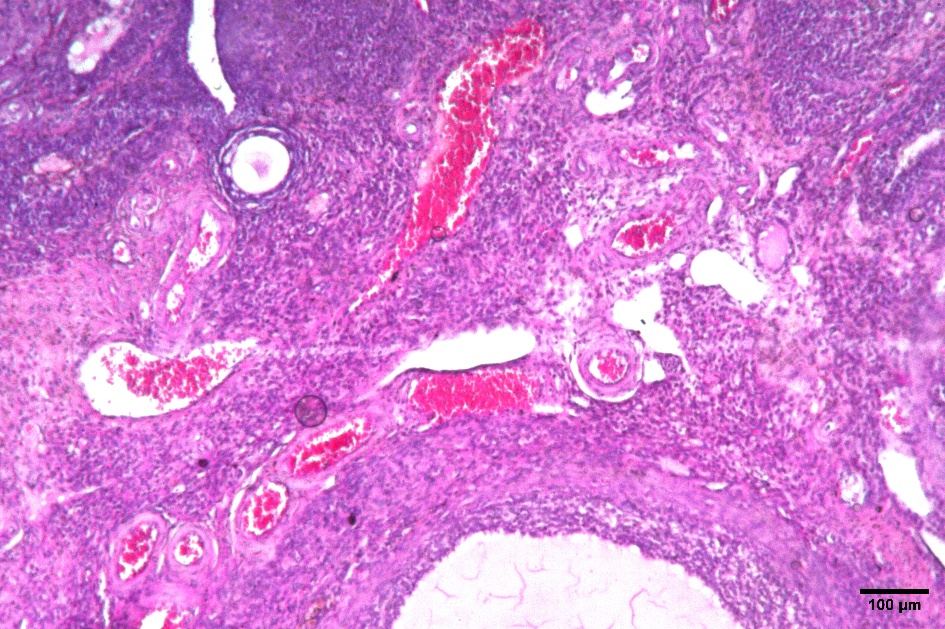

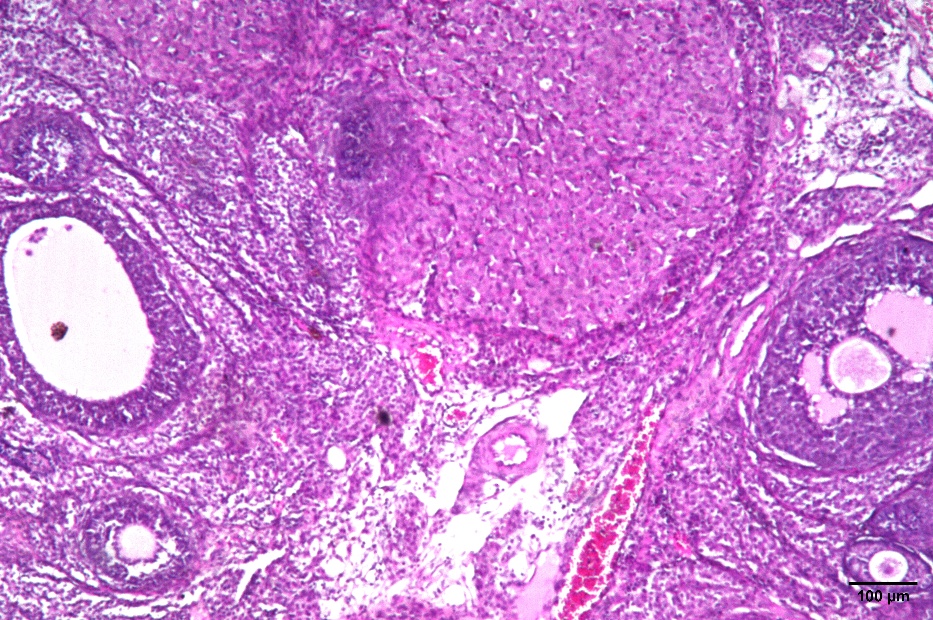

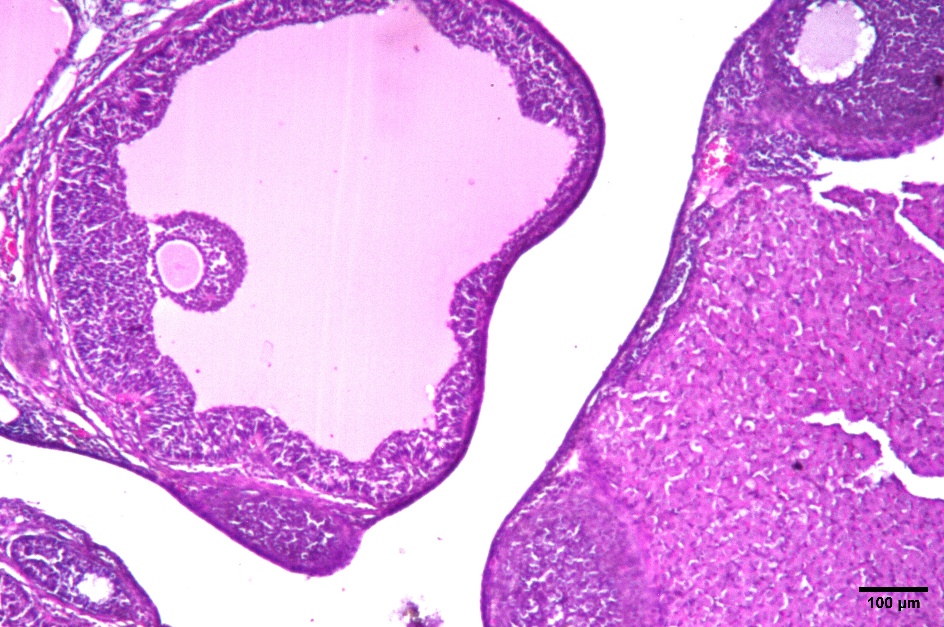

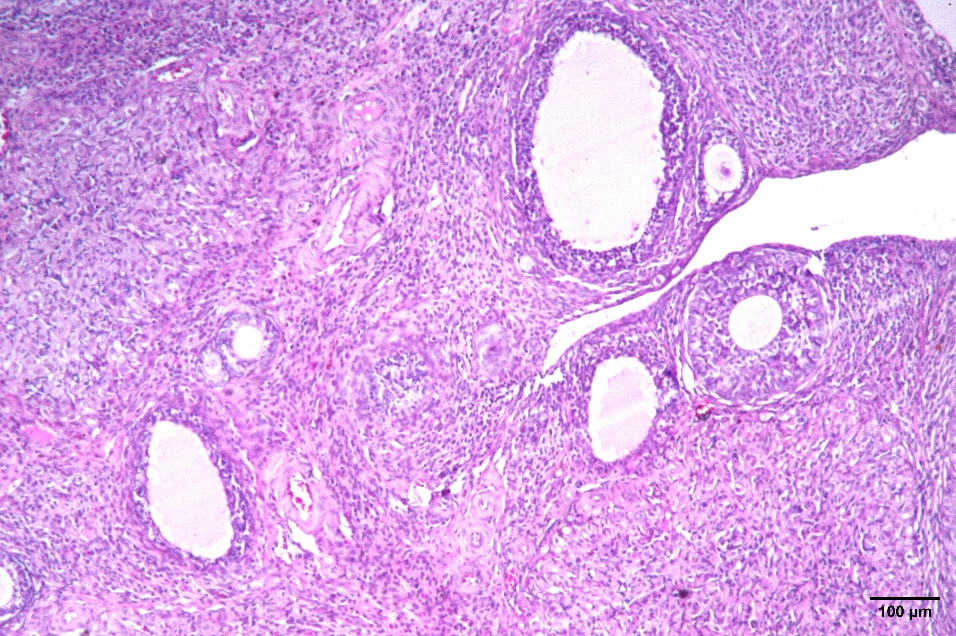

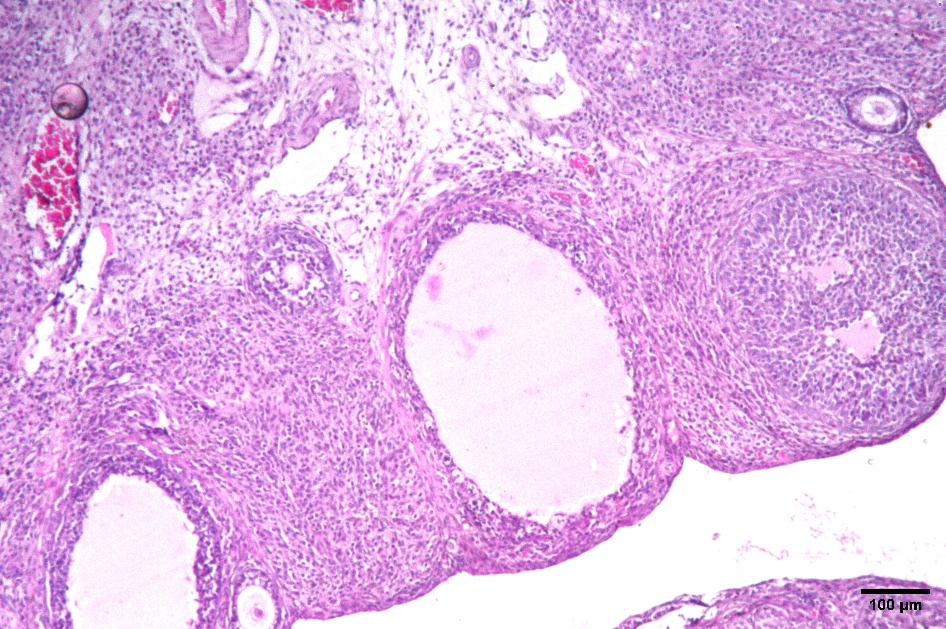

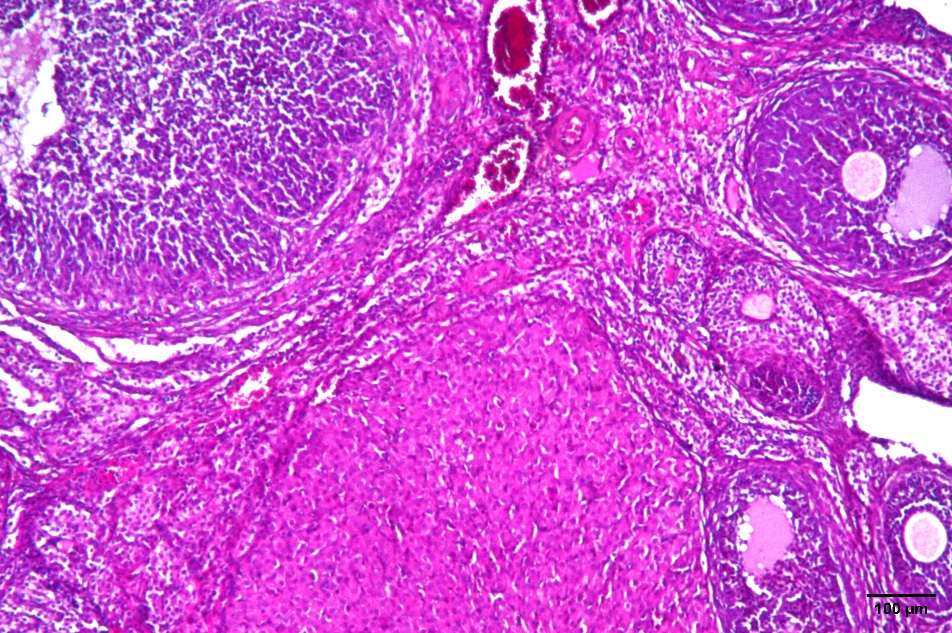

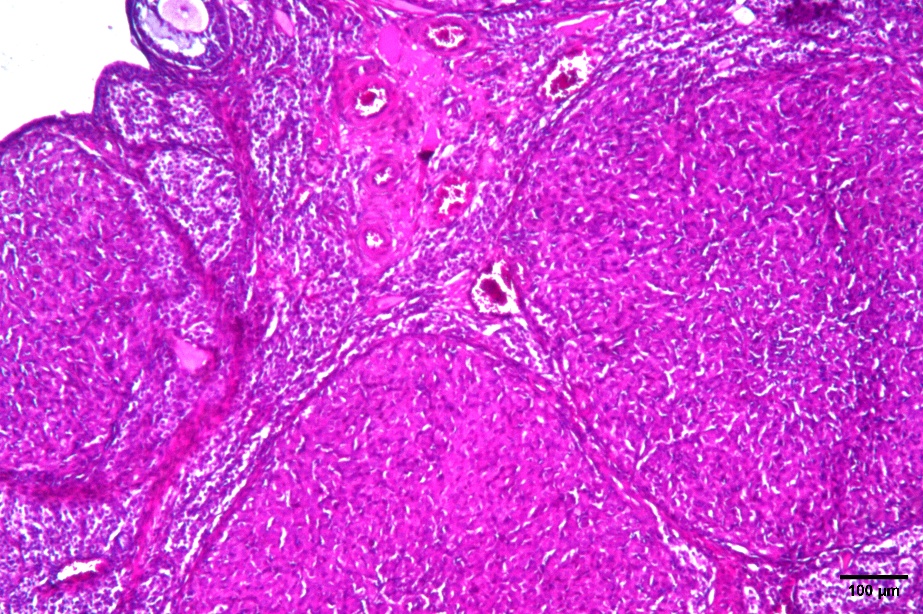


**A**

**B**

**C**

**D**

**E**

**F**

**G**

**H**


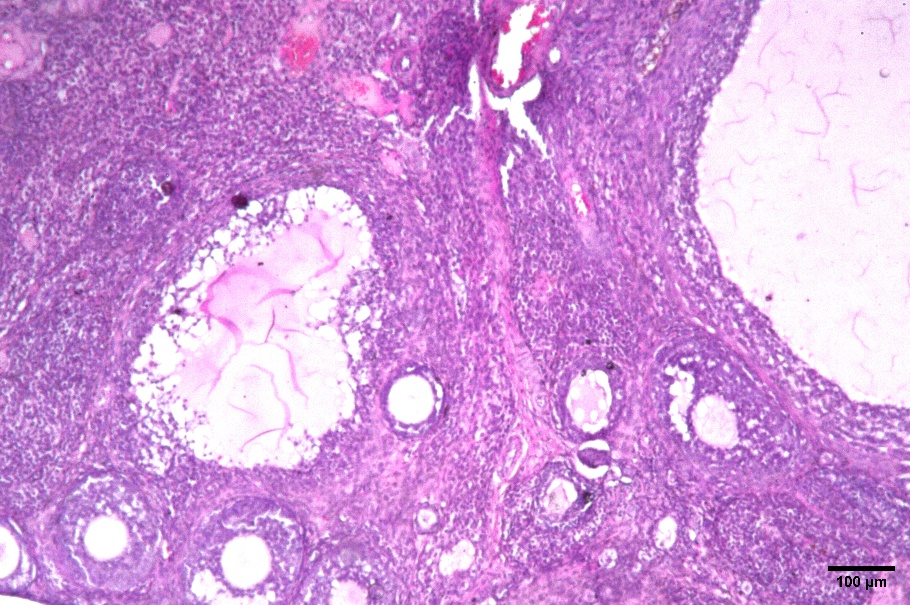


**Figure S5.** **Representative photomicrographs of ovarian sections stained with Hematoxylin and Eosin (H&E). (A & B)** LTZ + AVA 100 mg/kg group **(C & D)** CMC + AVA 300 mg/kg group **(E & F)** LTZ + AVA 300 mg/kg group **(G & H)** CMC + RSV 20 mg/kg group. *Scale bar = 100*µm.


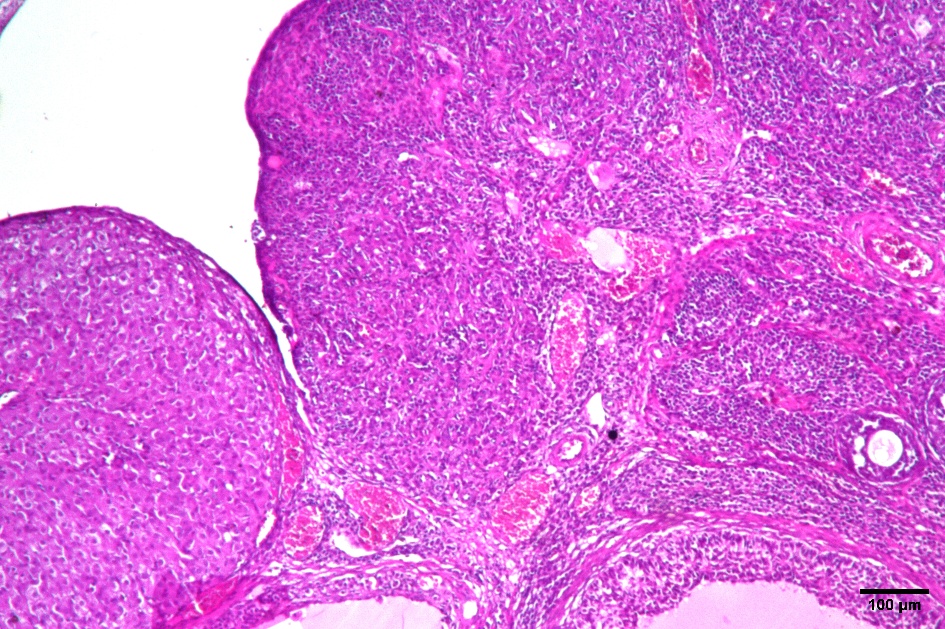


**A**

**B**


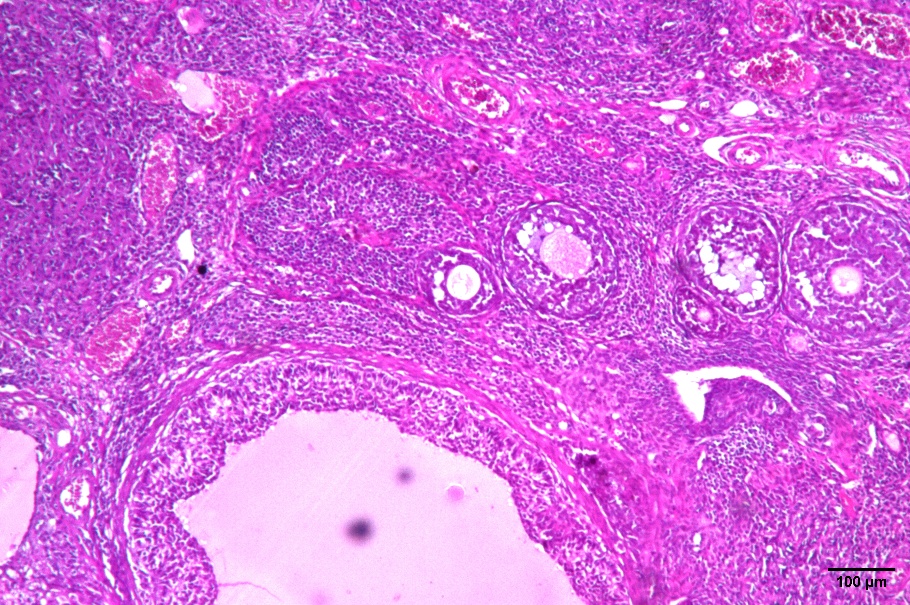


**Figure S6** **Representative photomicrographs of ovarian sections stained with Hematoxylin and Eosin (H&E). (A & B)** LTZ + RSV 20 mg/kg group. Scale bar = 100µm.


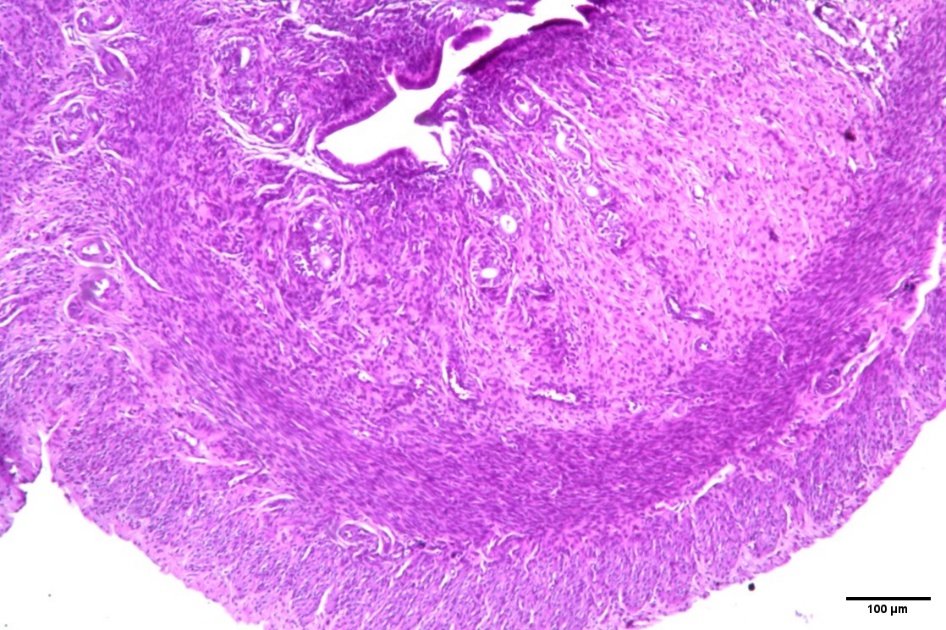

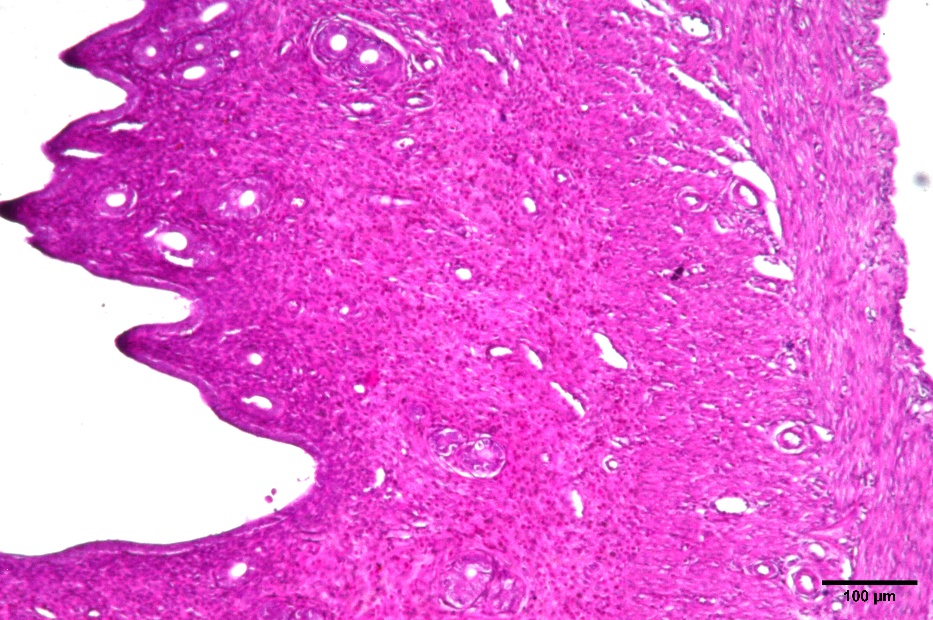

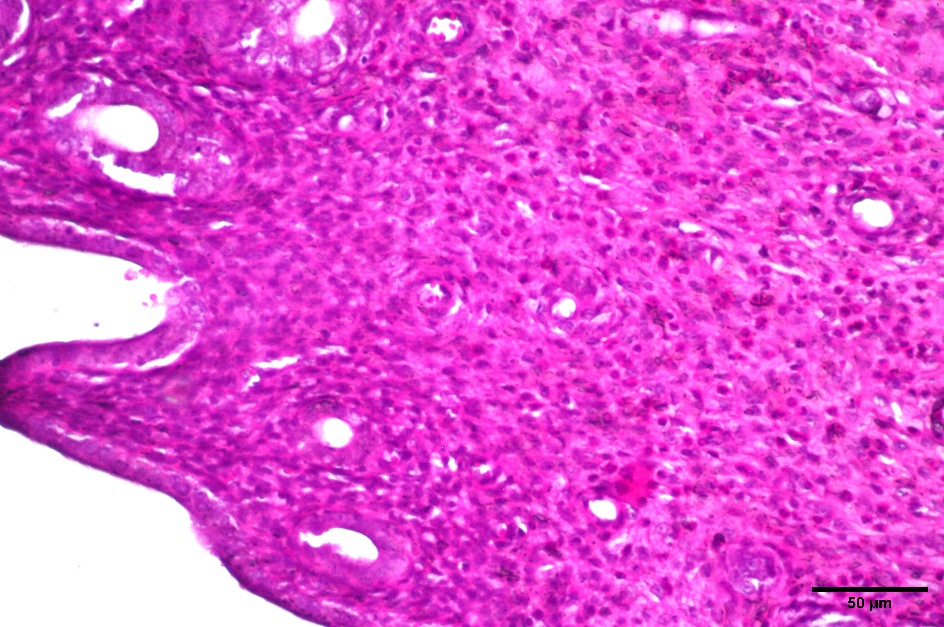

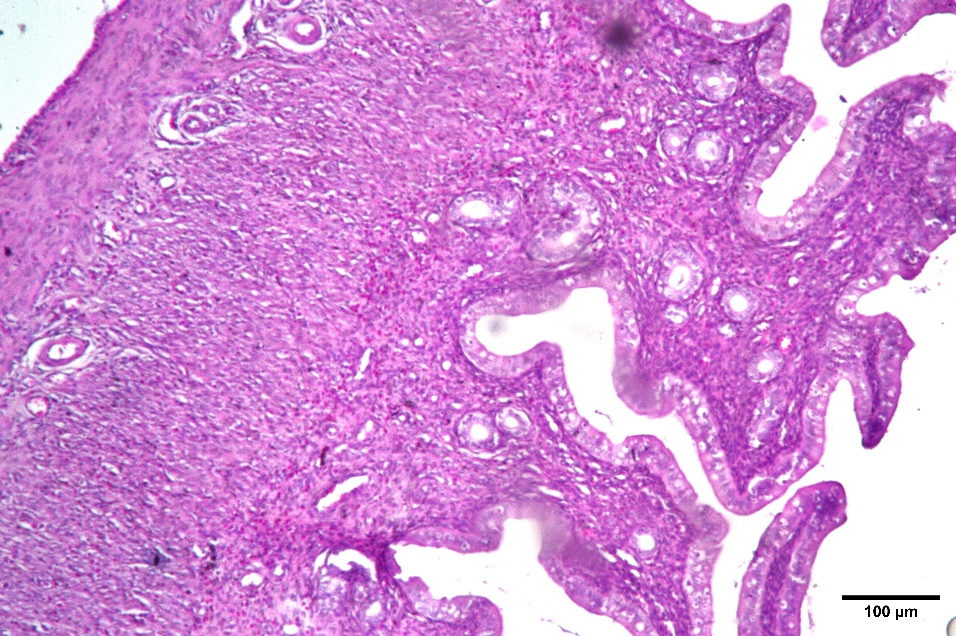

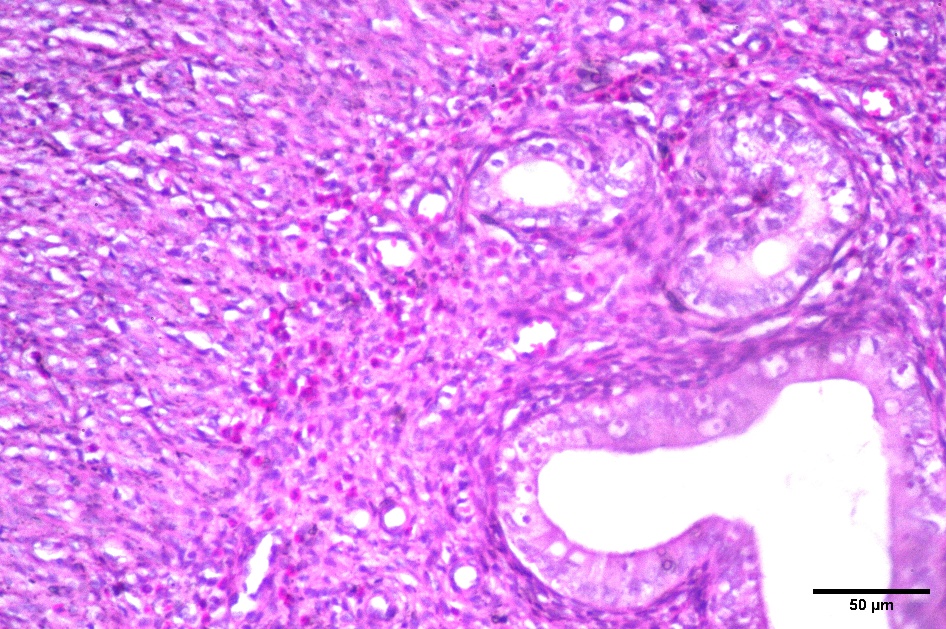

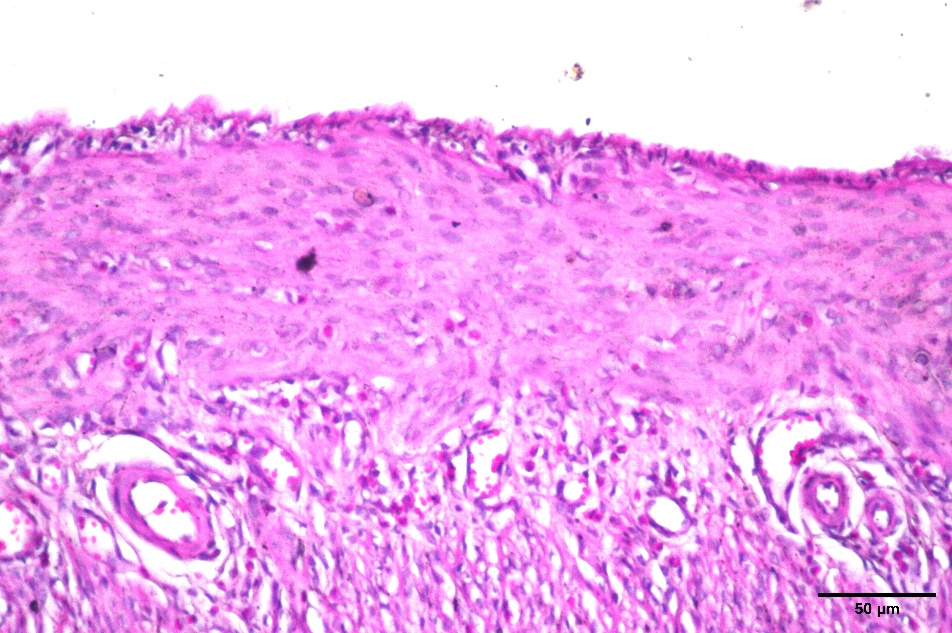

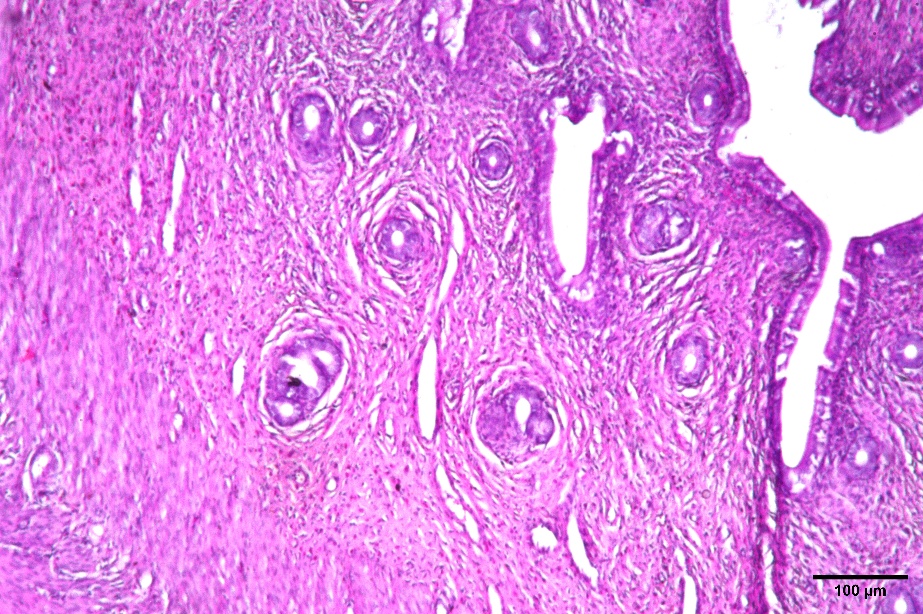


**A**

**B**

**C**

**D**

**E**

**F**

**G**

**H**


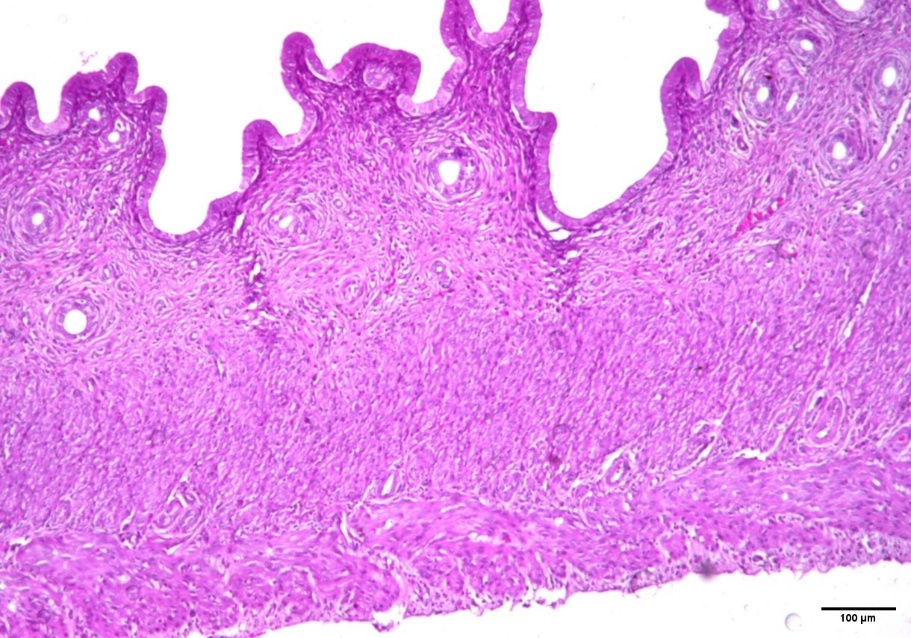


**Figure S7** **Representative photomicrographs of uterine sections stained with Hematoxylin and Eosin (H&E). (A)** Control (CMC) group (Magnification 20x, Scale bar = 100µm). **(B)** Letrozole (LTZ) group (Magnification 20x, Scale bar = 100µm). **(C)** CMC + AVA 100 mg/kg group (Magnification 20x, Scale bar = 100µm) **(D)** CMC + AVA 100 mg/kg group (Magnification 40x, Scale bar = 50µm) **(E)** LTZ + AVA 100 mg/kg group (Magnification 20x, Scale bar = 100µm) **(F & G)** LTZ + AVA 100 mg/kg group (Magnification 40x, Scale bar = 50µm) **(H)** CMC + AVA 300 mg/kg group (Magnification 20x, Scale bar = 100µm)


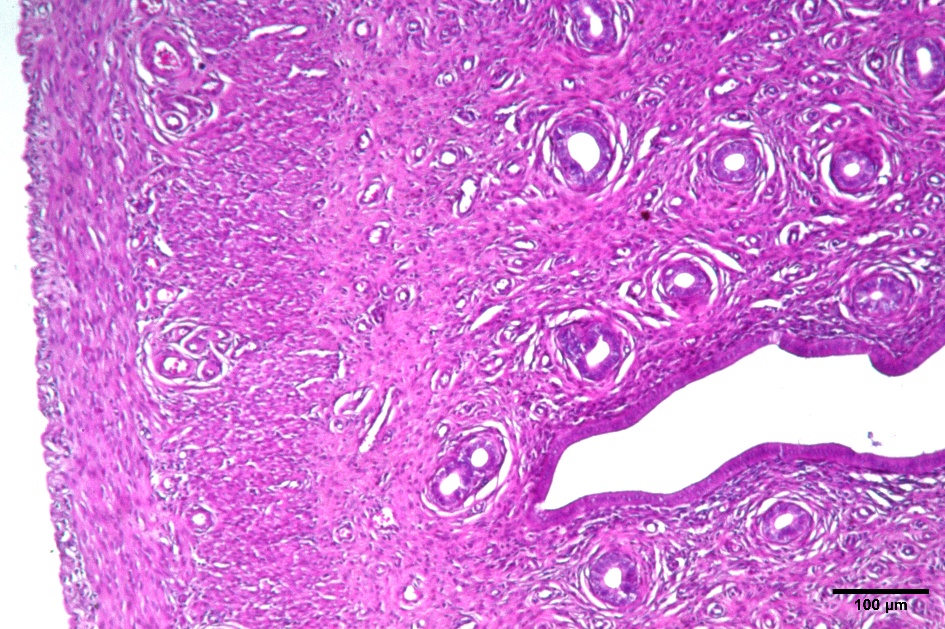

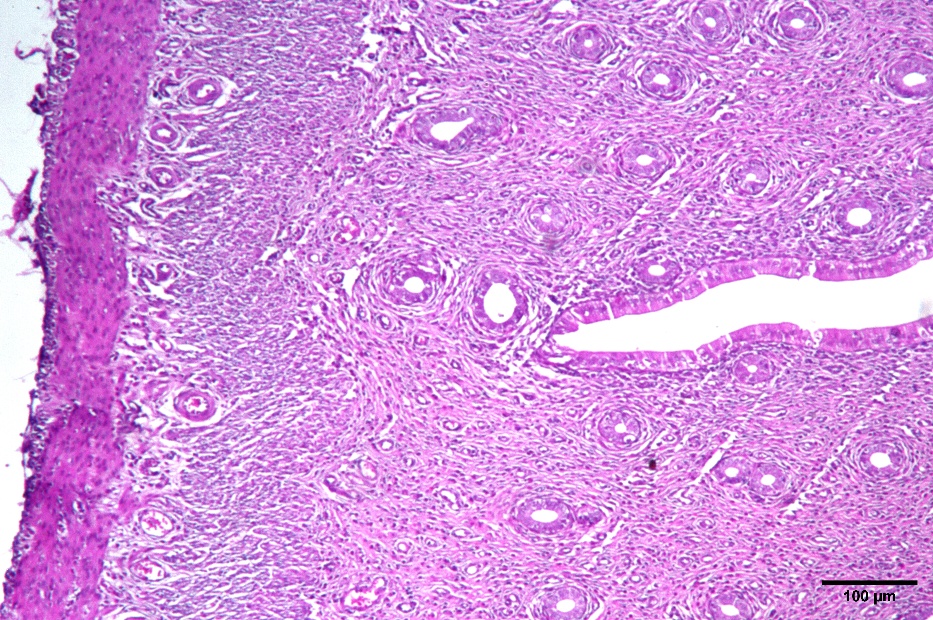


**A**

**B**

**C**


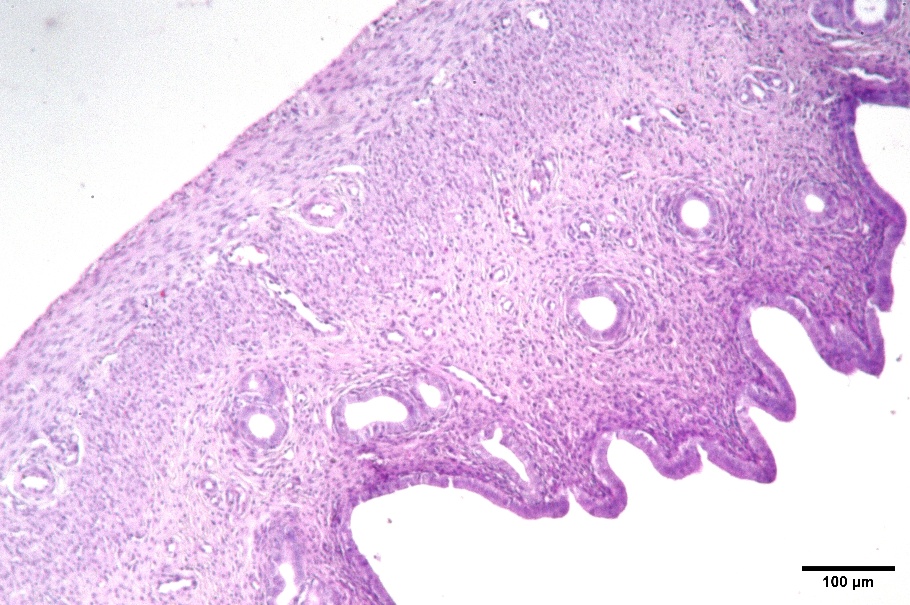


**Figure S8.** **Representative photomicrographs of uterine sections stained with Hematoxylin and Eosin (H&E). (A)** LTZ + AVA 300 mg/kg group **(B)** CMC + RSV 20 mg/kg group **(C)** LTZ + RSV 20 mg/kg group (Magnification 20x, Scale bar = 100µm).


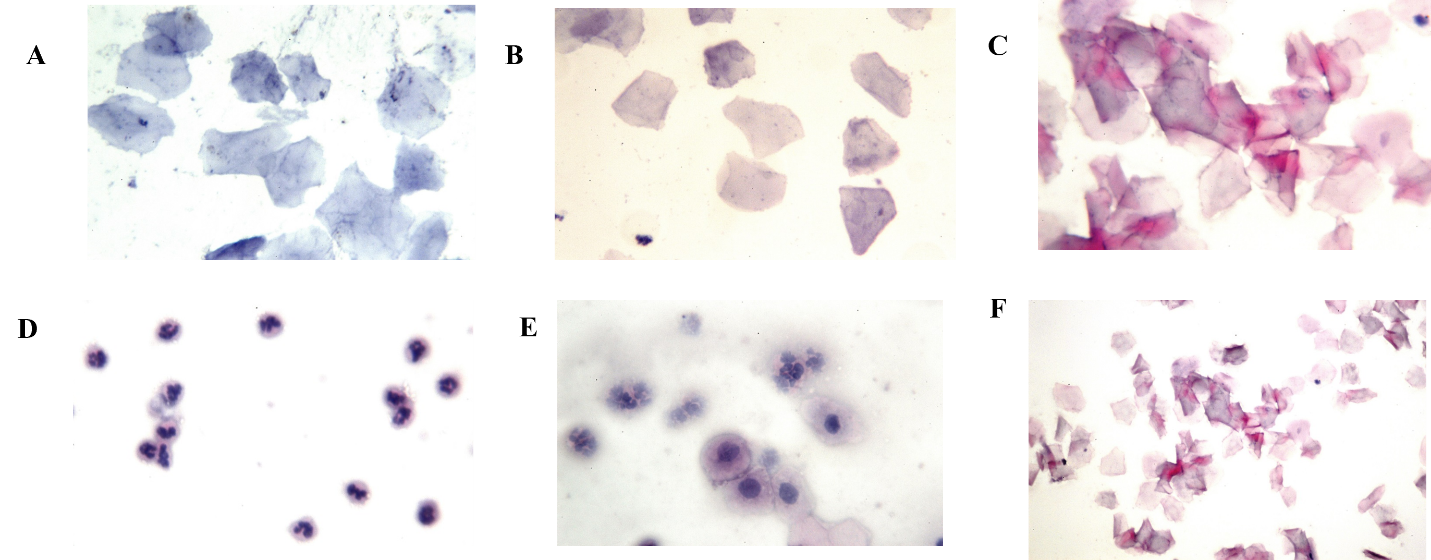


**Figure S9 Representative vaginal smear cytology illustrating estrous cycle patterns across experimental groups. (A, B & C)** Estrous phase characterized by abundant anucleated cornified epithelial cells seen in CMC Control group treated with only the vehicle, carboxymethyl cellulose (CMC), CMC + AVA 100 mg/kg group, CMC + AVA 300 mg/kg group, CMC + 20 mg/kg trans-resveratrol (RSV), and LTZ + RSV 20 mg/kg groups **(D)** Letrozole-induced PCOS rats showed persistent diestrus with predominant leukocytes **(E)** LTZ + AVA 100 mg/kg group displayed metestrus phase, evidenced by a mixed population of cornified epithelial cells and neutrophils, indicating partial restoration of cyclicity **(F)** Treatment with 300 mg/kg avenanthramides-enriched extract normalized the estrous cycle to estrus in PCOS rats. Scale bar = 25 µm.
